# Smart Nanosensor Technology for Fruit Preservation: *In vivo* Papaya Model with Ethylene Scavenging and Colorimetric Indication

**DOI:** 10.1021/acsomega.6c05286

**Published:** 2026-09-21

**Authors:** Ricardo dos Santos Medeiros, Ana Carolina Gava de Barros da Silveira, Giovanna Maia, Matheus Godoy Reimberg Amaral, Ramon Peres Brexó, Ednaldo José Ferreira, Elaine Cristina Paris, Marcos David Ferreira

**Affiliations:** † Institute of Chemistry (IQSC), University of São Paulo (USP), São Carlos, SP 13566-590, Brazil; ‡ Department of Chemistry, Federal University of São Carlos (UFSCar), São Carlos, SP 13565-595, Brazil; § 564899 Brazilian Agricultural Research Corporation, Embrapa Instrumentation, São Carlos, SP 13561-206, Brazil

## Abstract

A nanosilica-permanganate colorimetric nanosensor (NS) was developed for ethylene scavenging in postharvest papaya, offering sustainable shelf life extension. Incorporated in a polyethylene nonwoven pouch (tag), NS was compared to control (CO) and vermiculite (VR) treatments. NS particles measured 15–25 nm, negatively charged, and characterized by FTIR, SEM-FEG, SEM-EDS, and zeta potential. GC-FID analysis showed ethylene release at climacteric peak: CO = 0.50 μL/kgh, VR = 0.49 μL/kgh, NS = 0.35 μL/kgh, confirming NS’s superior scavenging. Ripening was monitored over 17 days at 16 °C. Deconvolution revealed ethylene peak at day 15 for NS vs day 13 for CO/VR. Firmness declined (peel: ∼30–6 N; pulp: ∼11–0.5 N), pH rose (5.4–5.9), titratable acidity (∼0.085–0.052%), and soluble solids rose (∼10.0–12.7). Statistical analysis did not demonstrate a significant difference among treatments. Hue angle in NS changed from 240° to 320° (violet to brown), indicating continued ethylene adsorption, unlike VR. NS color shift aligned with fruit ripening, supporting its use in smart packaging.

## Graphical abstract

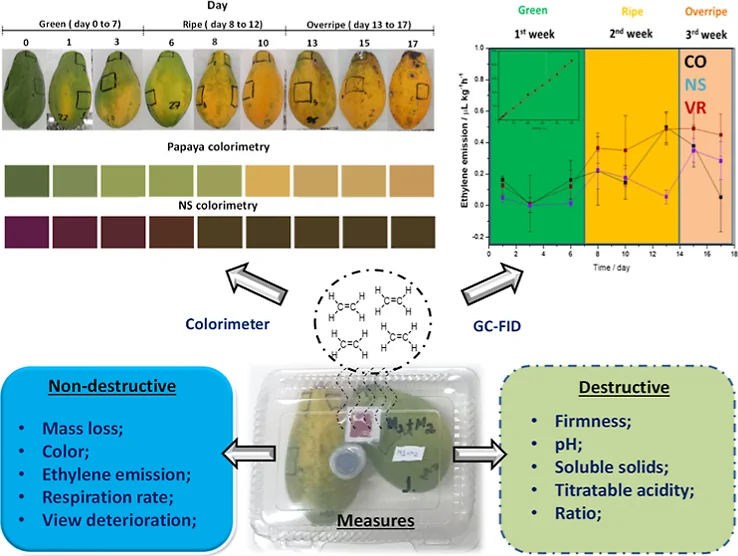

## 1 Introduction

According to the *Food Waste Index Report 2024*, approximately 1.05 billion tons of food (19% of the total produced) were wasted in 2022. Losses primarily occurred during storage, transportation, and processing stages, with postharvest to retail entry phases accounting for 13% of the total waste (UNEP, 2024). In the postharvest field, efforts are underway to develop new technologies that help reduce the waste of fruits and vegetables, as well as extend storage and shelf life.^1–3^

Smart packaging systems provide consumers with quality and safety information through signals that allow direct monitoring of the food system or its surrounding environment.^4^ In this context, technologies focused on the development of smart packaging with active compounds have been designed to capture gases emitted during fruit ripening.^5^ These compounds also work to control microbial contamination, reducing losses from processing to consumption by lowering the metabolic rates of fruits and vegetables. In addition, sachets containing inorganic, organic, or hybrid absorbent and adsorbent materials have been employed to control the production of the ripening hormone ethylene, consequently slowing fruit ripening.^1,6–13^

Ethylene is a hydrocarbon with the molecular formula C_2_H_4_, composed of carbon and hydrogen atoms. It has an unsaturated C=C bond and saturated C–H bonds, exhibiting planar geometry due to its sp2 hybridization. This molecule exists in a gaseous state, liquefying at −169 °C and boiling at −103 °C. Ethylene is a colorless, flammable gas with no odor.^14^ Fruits and vegetables are classified as either climacteric or nonclimacteric. Climacteric fruits continue to ripen after harvest, undergoing successive stages from green to overripe. These stages are directly associated with the fruit’s metabolic processes.^15^ Ethylene production is a key phase in fruit physiology, as it triggers biochemical reactions in various metabolic pathways, sustaining the ripening process.^16^ Therefore, understanding ethylene release patterns is crucial for controlling ripening.

According to the International Union of Pure and Applied Chemistry (IUPAC),^17^ chemical sensors are materials that convert chemical information into measurable signals. This information can come from a chemical reaction or changes in the physical property of the system being measured. Color is a fundamental property that serves as an indicator of the physicochemical state of biological samples. Accordingly, a colorimetric sensor is a device whose color changes in response to analyte interaction.^17,18^

On the other hand, an ethylene scavenger can be classified as absorber and adsorber. The gas interacts physically or chemically with composed matrices of inorganic/hybrid metals actively sprayed in micro and nanoscale. The most used material is KMnO_4_ (power oxidant agent) in a matrix of aluminosilicate, once it presents strong interaction with ethylene acting in double bond of molecules.^1^ Inorganic absorbers and adsorbers include materials such as silicas,^1,6,7^ alumino-silicate clays (vermiculite, montmorillonite, kaolinite),^1^ and zeolites.^12^ These materials can be combined with oxidizing agents like KMnO_4_ or other metals such as TiO_2_, Pd, Ag, Mo_7_O_24_, WO_3_ ^7,10,19^ to react with ethylene gas released during fruit ripening, which results in the effective delay of the ripening process. In addition, these matrices can also absorb or adsorb other gases like CO_2_, O_2_, and water vapor, which may be trapped within their packed lattice structures.^1^ Conversely, KMnO_4_-based sachets are widely used as ethylene adsorbers to extend fruit shelf life, though their color properties are seldom explored. Brackmann et al. and Wendt et al.^20–22^ observed reduced ethylene production and respiration in Gala, Royal Gala, Golden Delicious, and Brookfield apples. Nugroho et al.^23^ reported extended banana shelf life under controlled conditions, while Shahzad et al.^24^ and Ali et al.^25^ noted lower ethylene levels and delayed ripening in mangoes and tomatoes, respectively. Similarly, Silva et al.^26^ found that KMnO_4_ delayed the climacteric peak in Sunrise Golden papaya. More recently, Shin et al.^27^ developed a KMnO_4_-based colorimetric label for kiwifruit, correlating the visible color transition with ethylene adsorption, demonstrating its potential for smart packaging. Otherwise, applications employing potassium permanganate (KMnO_4_) to impart colorimetric properties to these materials for fruit ripening sensing remain scarce. Although several studies report the violet-to-brown transition associated with the reduction of Mn^7+^ to Mn^4+^ during ethylene oxidation,^5^ few establish a quantitative correlation between color change, ethylene adsorption, and fruit shelf life, resulting in largely qualitative assessments of the colorimetric behavior.

Nanomaterials have shown significant potential in postharvest technology. Their application in nonwoven fabrics (NWF) helps mitigate fruit and vegetable losses, directly supporting the United Nations’ Sustainable Development Goals (SDGs).^28^ Nanostructured materials often demonstrate superior efficiency when interacting with specific systems, primarily as a result of their increased surface area and reduced particle size.^29^ Consequently, nanomaterials have gained prominence and are increasingly being tested and investigated for postharvest losses.^3,30^ Coupled with its ethylene absorption and adsorption properties, permanganate-based nanosilica exhibits potential as an indicator of fruit ripening stages. However, a comprehensive understanding of the physicochemical characteristics of this nanomaterial, as well as its multifactorial interactions with ethylene, and fruit physiology, remains limited. In particular, the effects of such nanosensors on quality preservation, shelf life extension, and the ripening dynamics of climacteric fruits during postharvest storage especially papayas have not been fully elucidated. Therefore, sensor devices such as permanganate-based nanosilica tags should be evaluated not only from a metrological perspective but also in terms of their active role in modulating ripening progression. Few studies in literature have simultaneously addressed both these aspects, highlighting the dual and integrative approach of the present work as its primary scientific novelty.

This study aims to develop and evaluate a smart nanosensor system based on permanganate-impregnated nanosilica comparing to a commercial vermiculite-based ethylene absorber and a control group for fruit monitoring and preservation, using papaya as a highly perishable *in vivo* model. Postharvest chemical–physical parameters were evaluated on fruits during storage correlating sensor color changes with fruit maturity stages, integrating analytical validation with an assessment of its active role in modulating the ripening process. Therefore, this research contributes to the advancement of smart packaging technologies intended to mitigate postharvest losses of fruits and vegetables.

The nanosensor (NS) developed in this study was designed to perform a dual function, combining the chemical removal of ethylene through its oxidation by potassium permanganate with a visible colorimetric response. As ethylene released by the fruit reacts with KMnO_4_ supported on the nano-SiO_2_ matrix, the material progressively changes from violet to brown, providing a visual indication of the evolution of the scavenging process. Unlike previous studies that have predominantly evaluated either the colorimetric response of permanganate-based systems or the ethylene-scavenging capacity of inorganic matrices separately, the present study combines colorimetric monitoring with quantitative ethylene determination by GC-FID throughout fruit ripening. This approach enables the temporal relationship between ethylene production, NS color transformation, and fruit maturation to be evaluated. In addition, kinetic analysis and statistical treatment were incorporated to quantitatively describe the evolution of ethylene during storage, aspects that have received limited attention in previous studies of colorimetric ethylene scavenging systems. The NS was further incorporated into a tag placed in the package lid and evaluated directly during papaya storage, allowing its effect on fruit quality and shelf life to be assessed under practical storage conditions. Thus, the main contribution of this work is the integration of ethylene removal, visual indication, quantitative monitoring, kinetic analysis, and application in a real fruit-packaging system within a single experimental approach. The proposed strategy may also be adapted to other climacteric fruits after adjustment of the scavenging capacity according to their ethylene production.

## 2 Material and Methods

### 2.1 Chemical and Structural Sensor Materials

The silica (Tixosil 333) used in the nanosensor preparation was supplied by Rhodia Solvay Group, batch B9G20LINB1. It is an amorphous precipitated silica, with the particle size determined via Field Emission Scanning Electron Microscopy (FE-SEM). The KMnO_4_ used was purchased from ACS Científica (a Brazilian supplier of standardized raw materials), with 99% purity. The vermiculite was donated for this study, and its properties were verified through laboratory characterization prior to use. Distilled water, produced via a reverse osmosis system, was used for nanosensor preparation.

The prepared nanosensor and vermiculite materials were enclosed in custom-made pillow-type sachets, with one side composed of nonwoven fabrics NWF (Fitesa industry, polypropylene, 55 g m^–2^) to allow gas permeability, and the other side made of transparent plastic to enable colorimetric measurements. The sachet containing the permanganate-based nanosensing or vermiculite powders is hereafter referred to as a “tag” (Figure 1b I–II).

**Figure 1:**
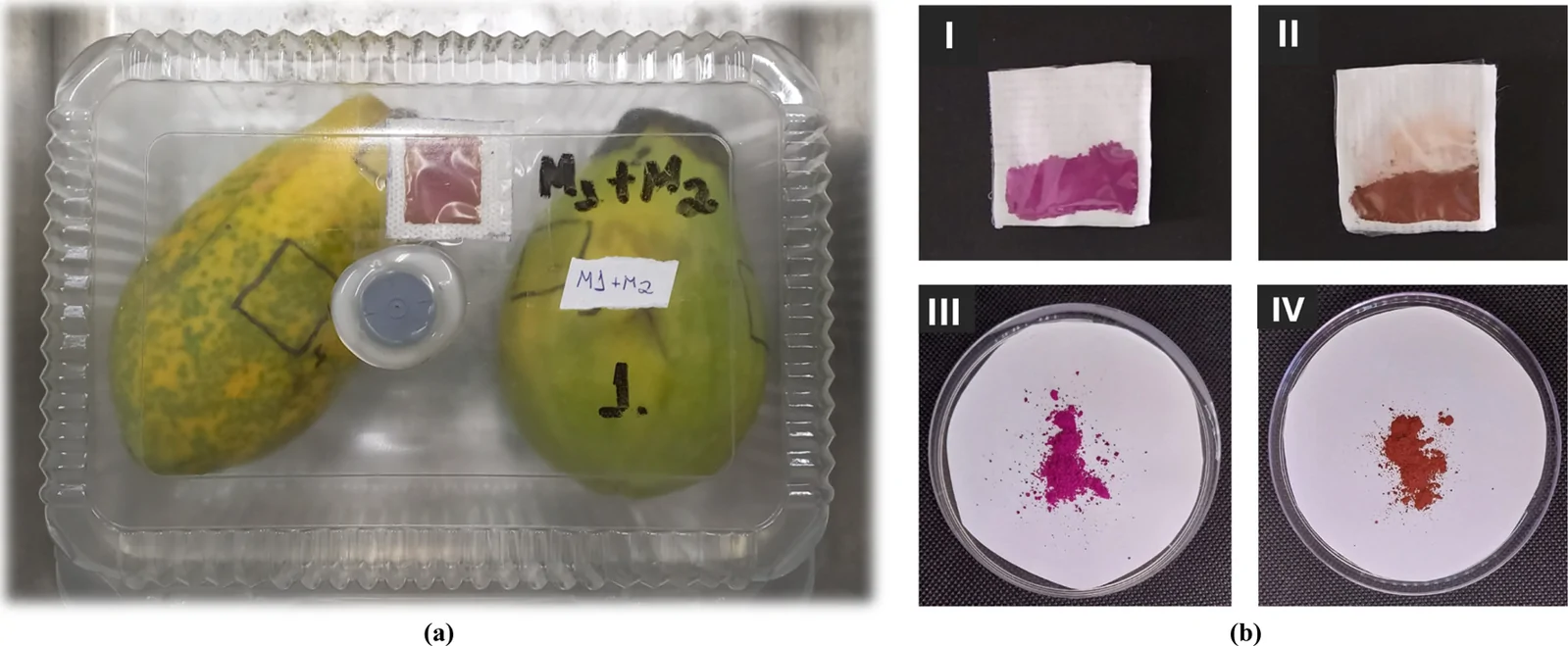
(A) Figure papaya in packing with nanosensor experimental setup. (b) Visual appearance of the pouch (tag) forms and powder of the (I, III) NS and (II, IV) VR. Image created by the authors.

### 2.2 Biological Materials

The papayas were acquired from Araquarara Terminal Market (CEASA), transported directly from a grower in Bahia state and delivered to the Postharvest Laboratory at Embrapa Instrumentation in São Carlos, SP, Brazil. Before being used in the experiment, all fruits underwent cleaning and sanitization processes, which included using neutral detergents and sodium hypochlorite solutions. The fruits were randomly selected to ensure uniformity among treatment groups at the same ripening stages for subsequent physical-chemical analyses. Fruit ripening stages were classified according to Bulsoto et al.,^31^ which defines six stages based on peel and pulp color. These fruits were examined and classified by peel characteristics. Of the fruits analyzed, an intermediary stage between 1 and 2 were observed, showing green peels with stripes less intense yellow in stage 1 and more intense in stage 2.^31^

### 2.3 Experimental Setup

The treatments were as follows: Control (CO), consisting of packaged papayas without any additive; Nanosensor (NS), consisting of packaged papayas with a nanosensor tag; and Vermiculite (VR), consisting of packaged papayas with a vermiculite tag. Polyethylene containers (21 × 15 × 12 cm; approximately 2.2 L) were used for fruit packaging. Each container featured a septum in the center of the lid to enable gas sampling. The nanosensor and vermiculite tags (NS and VR) were adhered to the inner surface of the lid to facilitate colorimetric readings. Two papayas were placed in each container, Figure 1a, simulating real-world shelf conditions. Fruits were stored for 17 days in a cold chamber at 16 °C and 50% relative humidity. The experimental setup was divided into two types of analysis: nondestructive and destructive. From this, there were separated five containers of such treatment CO, NS and VR for performed nondestructive analysis every 2 days totaling five replicates (n = 5), while the other containers were used for destructive analysis, held every 3 days.

### 2.4 Synthesis and Characterization of Materials

#### 2.4.1 Nanosensor and Vermiculite-Based Adsorber Synthesis

The nanosensor was prepared by weighing the masses of silica and KMnO_4_, adjusted according to the average volume released by the fruits. These materials were poured into a beaker, where 100 mL of H_2_O was added, and the mixture was subjected to stirring using a mechanical agitator under mild heating at approximately 50 °C. The system was stirred for 1 h, after which the material was transferred to a mortar and placed in an oven at 80 °C for drying, with the aim of preventing degradation. As the H_2_O evaporated, the material was ground with a pestle.^32^ Finally, the material was transferred to a Falcon tube for storage. The dried material exhibited a violet coloration, indicating its readiness for the intended application, Figure 1b III.

The synthesis of the ethylene absorber composed of the mineral vermiculite (VR) ((Mg,Fe,Al)_3_(Al,Si)_4_O_10_(OH)_2_·4H_2_O) and KMnO_4_ was carried out under the same conditions as the nanosensor synthesis, only varying the adsorbent matrix.^26,29,33^ The synthesized and dried composite exhibited a homogeneous texture and a terracotta color (Figure 1b IV).

Both syntheses were disposed of separately in tags and those placed individually inside the packages. The tags were prepared by cutting 3 × 3 cm squares from NWF and polyethylene sheets (Figure 1b I–II). Three sides were heat-sealed, 150 mg of either the NS or the commercial VR adsorbent was added, and the final side was sealed to form the tag. The prepared tags were stored under low-light conditions and in a controlled environment to preserve color stability and avoid premature reactions with humidity or gases that could affect the ethylene response. The final tag configuration with the absorbers is illustrated in Figure 1b.

#### 2.4.2 Zeta Potential

The NS measurements were carried out using a Zetasizer. A mass of powdered nanosensor material of approximately 2 mg was added to 20 mL of H_2_O and subjected to ultrasonic treatment for 13 min. Afterward, the suspension of the nanosensor was transferred using a Pasteur pipet to a specific cuvette for measurement. The measurements were performed in a triplicate of eight scans in the chamber of a microelectrophoresis instrument (Zetasizer Nano ZS ZEN3600, Malvern Instruments Ltd., Worcestershire UK).

#### 2.4.3 Fourier Transform Infrared Spectroscopy

Fourier Transform Infrared Spectroscopy (FTIR) measurements were conducted using a Bruker Vertex 70 RAM II spectrometer in ATR mode (equipped with an attenuated reflectance module attached to the laser line). The spectra were recorded in the range of 4000 to 400 cm^–1^ with a resolution of 4 cm^–1^ in 32 scans. Samples were predried in an oven at 80 °C for 30 min to remove absorbed water.

To evaluate the chemical stability of the NS, FTIR spectra were acquired immediately after synthesis and again after 30 days of storage under the same conditions adopted for the prepared tags. The spectra were recorded over the 400–4000 cm^–1^ spectral range and subsequently compared to assess possible chemical changes during storage.

#### 2.4.4 Scanning Electron Microscopy

Scanning electron microscopy (SEM) images were obtained using the following equipment: JEOL JSM6701F (Tokyo, Japan) for SEM-FEG (Field Emission Gun) measurements and JEOL JSM-6510 (Tokyo, Japan) for SEM-EDS (Energy Dispersive Spectroscopy) measurements. Sample preparation was performed by attaching carbon tape to clean stubs, depositing the material, and removing excess material by blowing. The samples were coated with platinum for SEM-FEG measurements and with carbon for SEM-EDS measurements. Using ImageJ software^34^ for particle and NWF analysis, their diameters and size porous average were found and illustrated in the histograms.

### 2.5 Postharvest Quality Analyses

Physical-chemical analyses were divided based on the nature of the measurements: nondestructive (weight loss, color, ethylene release, and respiration rates, severity index) and destructive (firmness, pH, titratable acidity, and soluble solids).

#### 2.5.1 Weight Loss

The weight loss was determined by weighing the fruit on the first day of analysis, experiment start, and on subsequent days until the end of the experiment for the three treatments (CO, NS, and VR). The weight loss percentage was calculated based on the difference between the initial and final weight values during the fruit ripening process.^35^

#### 2.5.2 pH and Titratable Acidity

The pH of the samples was measured using a Gehaka PG1800 digital pH meter, previously calibrated. The solution was prepared by weighing 10.0 g of homogenized fruit pulp into a 250 mL beaker, followed by the addition of 100 mL of distilled water. The solution was homogenized using a magnetic stirrer with a stirring bar. The pH meter electrode was immersed in the solution, and the pH was recorded once stabilization was achieved. Measurements were performed in triplicate for all treatments.

Titratable acidity (TA) was determined via potentiometric titration using a standardized 0.1 M NaOH solution, previously calibrated with potassium hydrogen phthalate. This solution was transferred to an amber flask and attached to a Jencons Digitrate Pro digital buret (50 mL). Titration was performed in the same solution used for pH measurements, with the equivalence point defined by a pH range of 8.2–8.4. Results were expressed in grams of citric acid per 100 g of sample, since it is the predominant organic acid in papaya.^36,37^ Titrations were performed in triplicate for all treatments, following the analytical standards of the Adolfo Lutz Institute.^38^

#### 2.5.3 Soluble Solids and Ratio

Total soluble solids (TSS) were measured using an Atago RX-5000cx digital refractometer (Tokyo, Japan), with results expressed in degrees Brix (°Brix). The analysis was carried out on pulp blended and homogenized using a Philco PMX700 Mixer, with all measurements performed in triplicate. The soluble solids-to-titratable acidity ratio represents the proportion of sugars to acids. This was calculated using the expression: Ratio (%) = TSS/TA.

#### 2.5.4 Firmness

Fruit firmness was evaluated by carrying out compressive tests based on the methodology described by Mishra et al.^33^ with modifications. A texturometer TA.XT plus Texture Analyzer (Stable Micro Systems Ltd., Godalming, UK), equipped with a 6 mm diameter probe at a penetration speed of 15 mm/s, penetration depth of 5 mm, and a contact area of 12 mm^2^ with the fruit peel. Overall firmness was measured at three angularly equidistant points on the equatorial plane of the fruit, directly on the peel, and results were expressed in Newtons (N) as mean values over time. For the pulp, six points were measured after cutting the fruit longitudinally along its major axis into two-halves: two in the peduncle region, two in the equatorial region, and two in the lower region. Firmness (in Newtons) was calculated from the slope of the force displacement curves for each sample.

#### 2.5.5 Colorimetry

Color parameters of both the papaya fruits and the tags (NS and VR) were measured using a Konica Minolta CR-400 colorimeter operating in the CIELAB system, which provides L* (lightness), a* (green-red axis), and b* (blue–yellow axis). Hue angle (h°), chroma (C*), and total color difference (Δ*E*) values were calculated according to Oliveira-Filho et al.^35^ and results were also converted to RGB format. Results are discussed in terms of light-color components (L*, C*, h°) and their association with other ripening parameters. Similarly to the firmness measurement procedure, fruits were measured at three equidistant points around the equatorial region, while the sensor readings were taken directly from the tag attached to the package.

#### 2.5.6 Calibration of the Nanosensor Colorimetric Response to Ethylene

The colorimetric response of the nanosensor (NS) to ethylene was evaluated under control conditions. Briefly, 150 mg of NS was placed in a sealed glass vial with a septum cap. Ethylene volumes of 27, 68, 135, 255, 322, 335, 349, and 363 μL were individually injected into the vial headspace using a gastight syringe. A control sample without ethylene was prepared under identical conditions.

The color change of the NS, resulting from its reaction with ethylene, was visually monitored. A direct correlation between ethylene concentration and the intensity of the color change was observed. Representative images were presented in the Supporting Information.

#### 2.5.7 Severity Index

The severity index was determined based on Oliveira Filho et al.,^35^ using a six-stage scale (0–100%) to assess fungal development and visual skin deterioration (darkening, wilting) throughout the storage period. Fruits were monitored for 17 days at 16 °C and 50% relative humidity in a cold chamber.

#### 2.5.8 Ethylene Release and Fruit Respiration Rate

Monitoring and quantification of ethylene gas were performed using a gas chromatography instrument, Agilent 8860 GC, equipped with Flame Ionization Detector (FID) and Thermal Conductivity Detector (TCD). The acquired data were processed and analyzed using Agilent’s Data Analysis software. A calibration curve ranging from 10 to 500 μL of ethylene using a 2.5-grade ethylene cylinder was fitted. The calibration process involved saturating a vial with pure ethylene, extracting aliquots within the pre-established range, and injecting them into separate vials. Each curve point is due to the volume injected, which was carried out in triplicate.

From the calibration curve, ethylene monitoring was conducted for fruit packaging. A preconcentration time of 4 h was set for instrument readings. Ethylene signals were observed at a retention time of 5.5 min, with their areas under the curve (integration) converted to units of μL/(kg.h) to analyze the profile and curve for each corresponding pair of fruits. The values obtained for release ethylene for all treatments were calculated.

The operating conditions for the ethylene quantification method was predefined as follows: run time of 9.4 min; ethylene peak observed at 5.5 min; injection of 300 μL of headspace gas collected via syringe through the sealing septum of the packaging; columns HP Plot Molsieve (30 m × 530 μm × 25 μm) and HP Plot Q (30 m × 530 μm × 40 μm); column flow rates of 10.0 mL/min (column 1) and 12.9 mL/min (column 2); carrier gases: argon, nitrogen, and hydrogen.

The respiration rate of the fruit was measured using an Illinois Instruments 6600 headspace oxygen/carbon dioxide analyzer, employing the same preconditioning used for gas chromatography-based ethylene evaluation.

### 2.6 Extracting Ethylene Emission Times during Papaya Ripening via Deconvolution

To obtain a more precise estimation of peak times, a deconvolution procedure was implemented in Python using a fitting algorithm based on two Gaussian curves. The algorithm requires input two initial estimates of the time positions of t_1_ and t_2_, which were derived from the mean ethylene response times of all papayas. The optimization is performed using the curve fitting methods provided by the Python library Scipy (scipy.optimize), allowing simultaneous adjustment of the parameters for both Gaussian functions. Fitting quality is assessed with the Pearson correlation coefficient. Thus, a suitable fit is identified as one with a higher correlation coefficient for both Gaussian curves. The final output includes the maximum values of both Gaussian curves and their corresponding time peaks (t_1_ and t_2_), which were targets of the statistical analysis. Figure 2 illustrates the deconvolution process, highlighting the estimation of t_1_ and t_2_.

**Figure 2:**
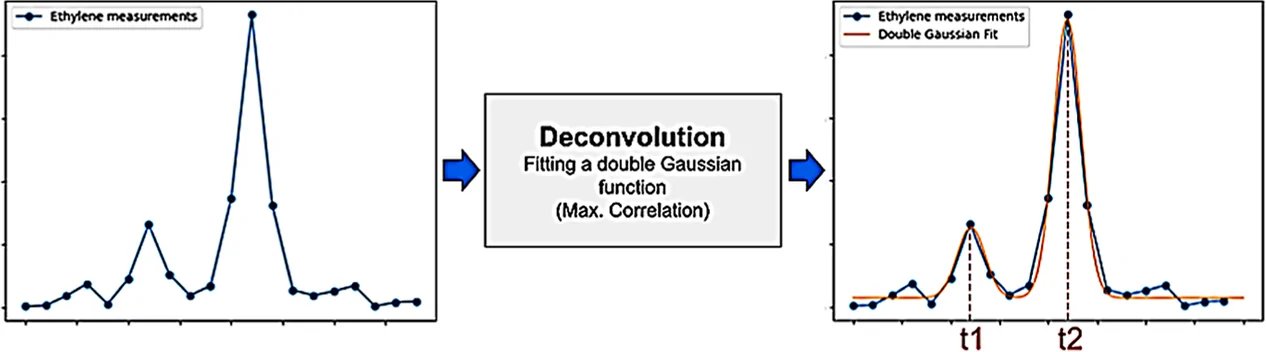
Determination of ethylene release timing (t_1_ and t_2_) by deconvolution analysis.

### 2.7 Statistical Analysis

For statistical analysis, a one-way ANOVA followed by Tukey’s post hoc pairwise comparison was performed to identify significant differences among treatments. The significance level was set at 5% (α = 0.05) for all tests. Statistical analyses were performed for all physicochemical parameters obtained from the destructive and nondestructive analyses, as well as for the ethylene release times (t_1_ and t_2_) obtained from the deconvolution process for each packaging, to assess the effects of the treatments on fruit ripening and shelf life extension.

For the color parameters, the sensitivity of the h°, L*, and C* curves were evaluated to infer the variation in fruit color as a function of the sensor color change. For all treatments, the calculations were made based on average values and standard deviation. For all other parameters and indicators evaluated, only descriptive statistics (means, standard deviation and graphs) were used.

## 3 Results and Discussion

### 3.1 Syntheses and Tag Setup

Commercially available sachets have been applied with this aim, although they lack colorimetric sensing functionality and act solely as absorbers and adsorbers.^1^ These sachets are typically made from inorganic matrices combined with specific percentages of KMnO_4_, an oxidizing agent that reacts with ethylene according to reactions (1–4)^28,39^

1 $$3{CH}_{2}{CH}_{2}\left(g\right)+2{KMnO}_{4}\left(s\right)+H_{2}O\left(l\right)\to 2{MnO}_{2}\left(s\right)+3{CH}_{2}CHO\left(g\right)+2KOH\left(aq\right)$$

2 $$3{CH}_{2}{CH}_{2}O\left(g\right)+2{KMnO}_{4}\left(s\right)+H_{2}O\left(l\right)\to 2{MnO}_{2}\left(s\right)+3{CH}_{2}COOH\left(g\right)+2KOH\left(aq\right)$$

3 $$3{CH}_{2}COOH\left(g\right)+8{KMnO}_{4}\left(s\right)\to 2{MnO}_{2}\left(s\right)+8KOH\left(aq\right)+6{CO}_{2}\left(g\right)+2H_{2}O\left(l\right)$$

4 $$3{CH}_{2}{CH}_{2}\left(g\right)+12{KMnO}_{4}\left(s\right)\to 12{MnO}_{2}\left(s\right)+12KOH\left(aq\right)+6{CO}_{2}\left(g\right)$$

A nanosensor based on nanosilica and KMnO_4_ was successfully synthesized via a wet-chemical route and compared with a commercial vermiculite-based ethylene absorber. The ethylene-scavenging performance and colorimetric response of both materials were investigated. The sensing mechanism is based on the reaction of ethylene with permanganate species (eqs 1–4), resulting in the reduction of Mn­(VII) to Mn­(IV) and the conversion of permanganate ions (MnO_4_^–^, violet) into manganese dioxide (MnO_2_, brown). This reaction produces a distinct color change that enables visual monitoring of ethylene exposure.^28,39^

Compared to commercial sachets, the developed tag offers two distinct advantages: (1) it maintains high ethylene permeability and absorption capacity, effectively delaying the ripening process; (2) its transparent polyethylene interface, Figure 1b I–II, enables real-time visual or machine monitoring of color transitions. Unlike conventional sachets, which only sequester ethylene without providing feedback, this tag performs a dual function: it serves as an active preservation agent while simultaneously providing a colorimetric correlation with the fruit’s ripening stage. This integrative functionality distinguishes a nanosensor based on this tag from current commercial alternatives.

A representative sample of the NS material was monitored over a 30 day storage period to evaluate its color stability prior to application. The NS remained stable throughout this period, exhibiting less than 3.3% variation in hue angle (Figure S1) and no visible color change, confirming that the premature reduction of MnO_4_^–^ could be considered negligible under the adopted storage conditions. Since KMnO_4_ is photosensitive and undergoes gradual photodegradation upon prolonged exposure to light, the nanostructured material was stored in sealed tubes under controlled low-light conditions during tag fabrication and throughout the experimental period.

### 3.2 Characterization

The following sections present the characterization results of the structural features of individual molecules and the composition of molecular mixtures.

#### 3.2.1 Zeta Potential

Zeta potential analysis revealed that the nanosensor exhibited a negatively charged surface, with a potential of −44 mV, indicating good colloidal stability. The hydrated radius showed a variable distribution, with the main peak centered around 131 nm. These results confirm the presence of a stable nanosensor dispersion with well-defined particle size distribution.

#### 3.2.2 Fourier Transform Infrared Spectroscopy

The FTIR spectra of SiO_2_, KMnO_4_, NS, and the tag component material were analyzed to identify the characteristic vibrational bands of the compounds. The oxidizing agent exhibited a band at 866 cm^–1^ corresponding to the Mn–O stretching vibration, while silica showed bands at 1061 cm^–1^, attributed to the O–Si–O vibration, and at 795 cm^–1^ associated with Si–O stretching. The NS sample presented bands characteristic of both nanosilica and the MnO_4_^–^ ion. Notably, the MnO_4_^–^ band shifted to 914 cm^–1^, which can be attributed to interactions with silanol groups.^40,41^ Additional FTIR spectra and details are provided in the Supporting Information (Figure S2a).

The tag material was analyzed to verify the groups present in the NWF with different granulometry on both sides and the plastic. Qualitatively, when comparing the experimental spectra with literature, it can be observed that the profiles spectra FTIR (Figure S2b) refer to the polymers, polyethylene and polypropylene, which are the material’s constituents used in the manufacture of the tag. The vibrations present in polyethylene are attributed to the C–H bonds at 2923, 2845 cm^–1^, at 1464 cm^–1^ referring to the stretching of the C–C group of the chain, as well as the bands of these bonds in polypropylene were observed at 2961, 2910, 2845, 1464, and 1373 cm^–1^.^42,43^

The FTIR spectra obtained immediately after synthesis and after 30 days of storage exhibited a nearly complete overlap throughout the 400–4000 cm^–1^ spectral range (Figure S3). This result indicates that no detectable chemical changes occurred during storage, suggesting that the KMnO_4_ remained chemically stable under the adopted storage conditions.

#### 3.2.3 FEG-SEM and SEM-EDS Imaging Analyses

FEG-SEM provided detailed visualization of the material’s size and morphology, Figure 3a. It also enabled analysis of the nonwoven’s pore size and the average fiber diameter, Figure 3b and c.

**Figure 3:**
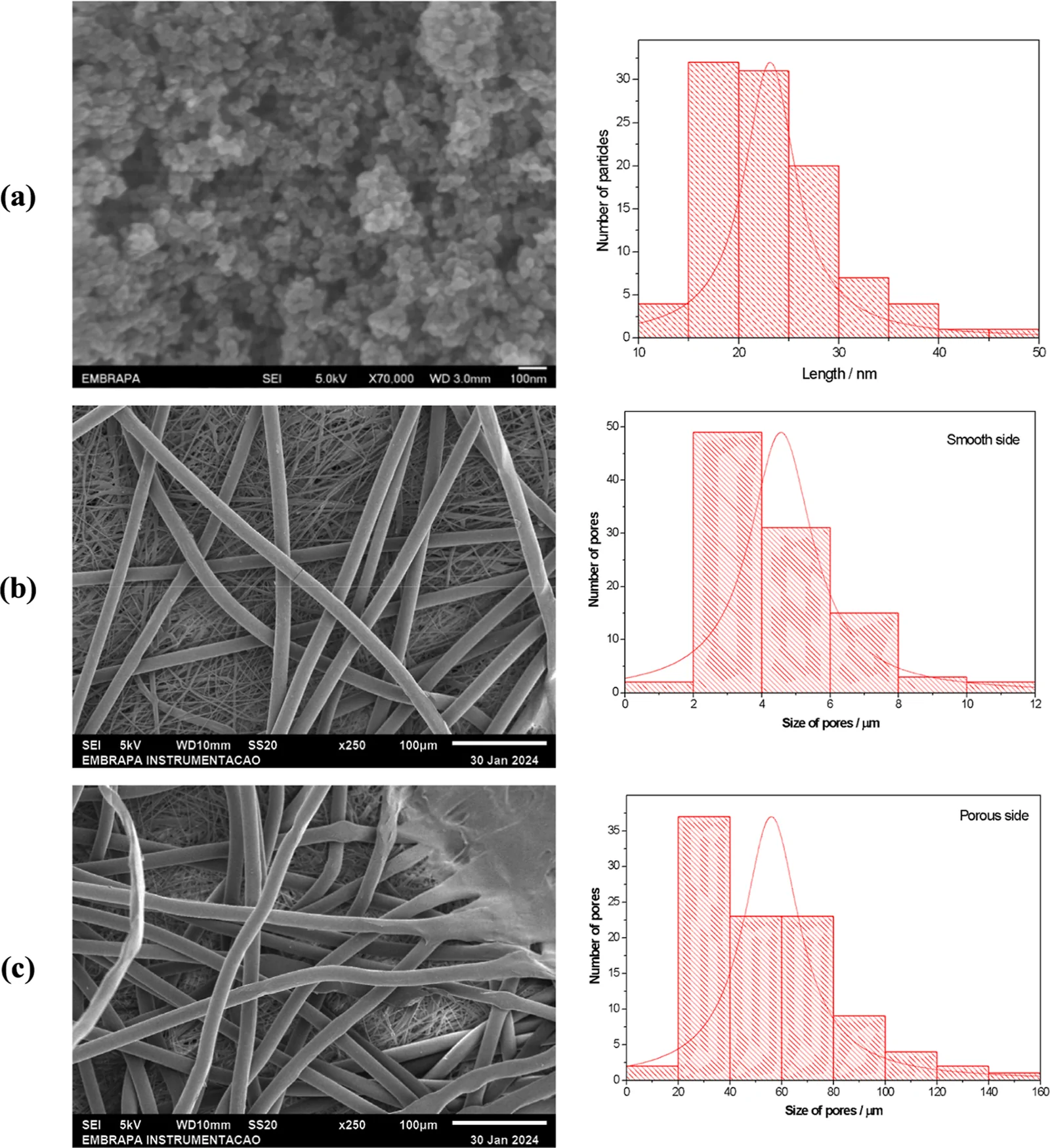
Scanning electron microscopy images. (a) Nanosensor and particle size counting histogram; (b) NWF smooth side and histogram; (c) NWF porous side and histogram.

Figure 3a shows that the NS sample synthesis, after drying and processing, results in irregularly spherical particles with average size to range 15–25 nm, as illustrated in the histogram. Figure 3b and c show electron microscopy images of the internal (porous) and external (smooth) sides of the NWF, respectively. The randomly intertwined fibers vary in diameter and pore size. On the porous side, thicker fibers (15–17 μm) create pores ranging from 10 to 150 μm. In contrast, the smooth side has thinner, more densely packed fibers (0.5–3.0 μm), resulting in smaller pores from 1 to 16 μm. This structural difference is crucial, as the material must prevent the passage of the inorganic absorber/adsorbent compound while allowing ethylene gas to diffuse for adsorption and reaction. However, the NS naturally tends to form particle clusters, which remain within the pore network. This ensures the tag’s active mass stays retained without passing through the material.

Regarding food-contact safety, the NS was designed as a tag attached to the packaging lid, operating exclusively in the package headspace without direct contact with the food matrix. Each tag contained 22.5 mg of KMnO_4_ as the active oxidizing phase. SEM analysis revealed that the nanostructured material formed agglomerates larger than approximately 17 μm (Figure 3a), whereas the NWF exhibited pore sizes ranging from approximately 2 to 12 μm (Figure 3b). This size difference provides physical confinement of the active material within the NWF, reducing the likelihood of particle release during storage. For future commercial applications, additional physical barrier strategies may also be incorporated to further minimize any potential contact between the active material and the food. Nevertheless, migration, leaching, and toxicological assessments were beyond the scope of this study. Accordingly, comprehensive food-contact safety evaluations, including migration testing and compliance with FDA and EFSA regulatory requirements, will be required prior to commercial implementation.

Additionally, SEM-EDS analysis was conducted to confirm the NS elemental composition and ensure that no contamination occurred during the synthesis. Figure 4a presents the elemental mapping and corresponding EDS spectra of the material. Figure 4b shows that silica is the main component of the inorganic matrix, with strong silicon and oxygen signals corresponding to silanol groups. Manganese from the permanganate is randomly distributed, along with its potassium counterion. Other elements detected are likely unrelated to the sample and appear at low intensity, except for carbon from the coating.

**Figure 4:**
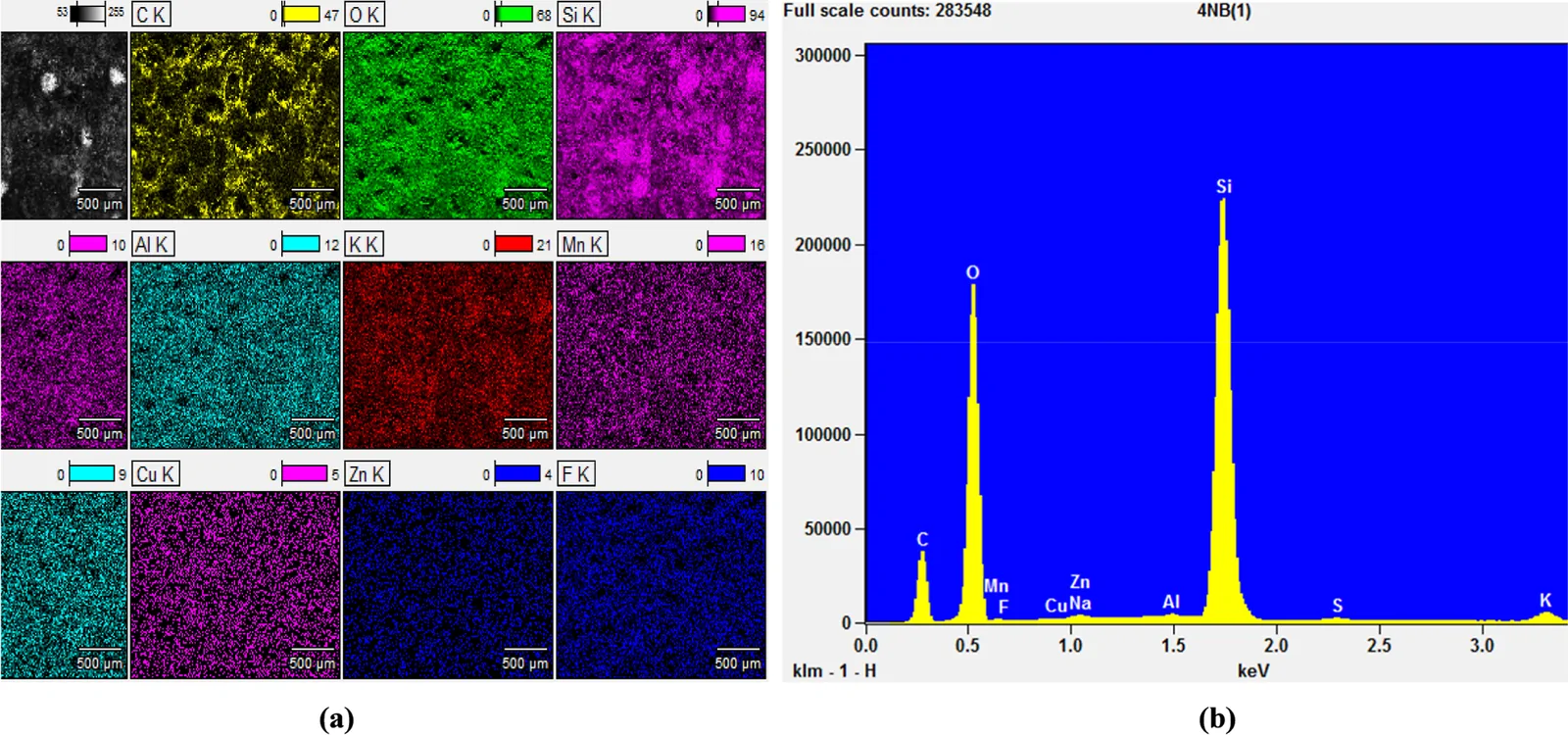
(A) Electron microscopy images with energy-dispersive spectroscopy detection (SEM-EDS) and (b) EDS NS spectrum.

#### 3.2.4 Postharvest Quality Analyses

##### 3.2.4.1 Physical-Chemical Analyses: Weight Loss, pH, Titratable Acidity, Soluble Solids and Ratio, Firmness, Severity Index

The control group (CO) experienced the highest average weight loss (4.99%) after 17 days, followed by the vermiculite group (4.87%), while the sensor group had the lowest (4.14%), (Figure S4). In the first week, the vermiculite group (VR) showed the greatest loss (1.09%), whereas the sensor group had the least (0.56%). This weight loss is primarily due to water loss through transpiration during ripening.^35^ One-way ANOVA performed for each evaluation week showed that the p-value (0.94) exceeded the significance level (α = 0.05). These results indicate that no statistically significant differences were observed among the groups (Table S4).

Table 1 presents the results for pH, TA, TSS, and the TSS/TA. Over 17 days, pH values increased, aligning with reported values for papaya (5.5–5.9). The TSS content rose due to ongoing hydrolysis and sugar formation (sucrose and hexose synthesis), intensified by water loss during ripening.^35,36,38,44,49^

**Table 1:** Results Means of pH, TSS, TA and TSS/TA of Papaya Over Time (17 days)

analysis	pH			TSS			TA			TSS/TA		
days	CO ±^a^SD	NS ± SD	VR ± SD	CO ± SD	NS ± SD	VR ± SD	CO ± SD	NS ± SD	VR ± SD	CO ± SD	NS ± SD	VR ± SD
1	5.46	5.42	5.56	10.09	11.28	8.45	0.078	0.086	0.051	128.55 ± 10.9	131.16	166.17
	±0.08	±0.06	±0.14	±3.06	±0.92	±3.26	±0.008	±0.013	±0.022		±6.36	±21.0
**3**	5.38	5.37	5.33	12.68	11.97	11.49	0.076	0.082	0.081	166.81	146.66	142.47
	±0.12	±0.06	±0.09	±0.50	±0.90	±2.50	±0.013	±0.015	±0.011	±11.7	±12.8	±15.9
**6**	5.40	5.69	5.64	12.45	11.45	12.48	0.071	0.060	0.065	175.33	190.53	192.76
	±0.06	±0.15	±0.15	±0.79	±1.29	±1.41	±0.010	±0.013	±0.019	±11.1	±14.1	±16.0
**10**	5.59	5.36	5.77	12.60	11.74	11.37	0.074	0.075	0.048	171.19	156.41	238.88
	±0.20	±0.5	±0.18	±1.24	±1.76	±1.80	±0.012	±0.042	±0.008	±10.9	±7.73	±9.99
**13**	5.74	5.91	5.73	11.90	11.66	9.94	0.068	0.053	0.060	174.74	217.87	165.24
	±0.17	±0.22	±0.12	±0.53	±0.77	±2.53	±0.016	±0.013	±0.010	±10.7	±17.2	±9.99
**17**	5.78	5.60	5.75	10.40	11.30	11.96	0.061	0.072	0.052	169.68	157.73	228.37
	±0.17	±0.39	±0.14	±1.51	±1.68	±1.25	±0.011	±0.036	±0.011	±25.7	±10.8	±11.6

a SD: standard deviation.

The sugar/acid ratio increased with ripening, indicating enhanced sweetness and reduced acidity traits preferred by consumers. This is attributed to gluconeogenesis, polysaccharide hydrolysis, and sugar accumulation during ripening.^35^ The ANOVA revealed no statistically significant differences for pH, TA, TSS, and TSS/TA ratio among treatments throughout the entire experimental period, with p-values >0.05 (Table S4 presents results p-values for such analysis).

No significant differences were found between treatments over the experimental period for firmness assay, with the p-values >0.05 (Table S4). However, it was observed that a general decrease in firmness was observed across all treatments (CO, NS, VR), consistent with fruit ripening, which weakens the cell walls of both peel and pulp (Table S1 and Figure S5). Initially, peel firmness ranged from 25 to 30 N and pulp firmness from 5 to 11 N. By the end of the experiment, these values dropped to 6–11 N for the peel and 0.5–1.6 N for the pulp.

In terms of trends over time, the NS group maintained greater peel firmness than the control up to day 10, suggesting delayed ripening due to ethylene capture by the NS, which may slow down metabolic processes. However, after day 10, firmness declined more rapidly in the NS group, indicating progression into ripening. The VR group exhibited firmness patterns like the control but remained firmer until day 13. Pulp firmness followed a similar trend across all groups: relatively stable until day 10, followed by a marked decline. These patterns reflect the transition from green-ripe to overripe stages, with decreasing structural integrity over time.

When analyzing the severity levels (Table S2) during the first week, fruits across all treatments exhibited low severity levels (indices 0 and 1), indicating either no deterioration or mild skin damage (up to 20%). However, by the third week, the control group showed advanced deterioration, with fruits reaching severity indices 3, 4, and 5. In contrast, fruits treated with the NS and VR exhibited delayed progression of ripening and appearance of diseases on the fruit skins, with several still at indices 1 and 2, indicating better preservation. Moreover, this suggests that the NS’s ethylene adsorption may have slowed ripening and extended shelf life. The trend is visually supported by the discussion in the next section and the color-coded data (Table S3).

The ANOVA statistical analysis results of treatment CO, NS and VR relate to measures mass, pH, TA, TSS, Ratio, Firmness were organizes and presented in Supporting Information (Table S4), allowing the observation of p-values results upper significance level (results >0.05), highlighting that there were not significant differences.

##### 3.2.4.2 Colorimetry

Fruit color evolution for CO, NS, and VR treatments was quantified through hue angle variation (Figure 5a) and correlated with the visual response of the indicator tags (Figure 5b). All fruits exhibited a decrease in hue angle from ∼115° to ∼90° by day 8, corresponding to the transition from green to yellow peel and coinciding with the initial ethylene peak, consistent with typical climacteric behavior reported for papaya.^49^ During this stage, the NS tag displayed a distinct shift from 240° to 280° (violet to magenta), indicating active ethylene interaction, whereas the VR tag remained essentially unchanged (∼265°), demonstrating limited responsiveness. Linear regression analysis of the hue angle was done, showed an excellent fit, with coefficient of determination (R^2^) equal the 0.939, indicating a pronounced colorimetric response of NS (Figure S6a), beyond analytical parameters presented in Supporting Information (Table S5).

**Figure 5:**
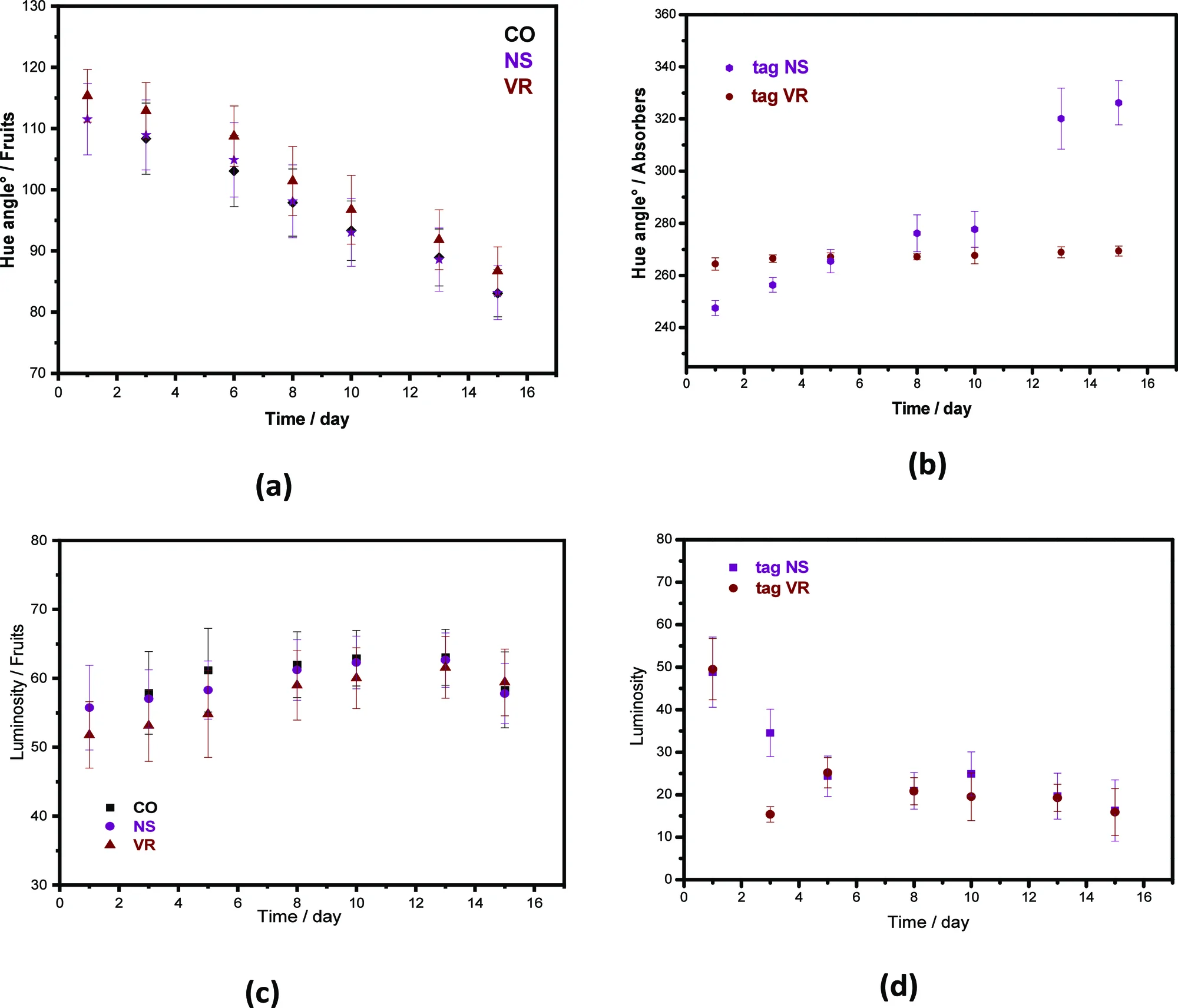
Hue angle curves as a function of time for fruits: treatments CO (black); NS (violet); VR (wine) (a) and tags NS and VR (b); Luminosity curves fruits (c) and tags (d).

Beyond day 8, fruit hue values approached 90°, reaching the climacteric peak around day 13, with CO and NS treatments falling below this threshold. Notably, the NS tag continued to evolve, reaching ∼320°, while VR remained stable (∼270°), corroborating the higher sensitivity and dynamic response of NS. This behavior is attributed to the progressive reduction of permanganate (MnO_4_^–^ to MnO_2_), enabling sustained ethylene scavenging and continuous optical response. Similar mechanisms have been reported for KMnO_4_-based systems, in which oxidation of ethylene delays ripening and extends shelf life.^50^ The temporal alignment between NS color evolution and fruit ripening highlights its dual function as both an ethylene absorber and a real-time ripening indicator, in agreement with previous studies on active packaging systems.^51^

The analysis of lightness (L*) complemented the hue angle results by providing additional information on the color evolution of both the fruit and the tags. Fruit L* increased from 51.8 to 63.5 up to day 13, reflecting peel brightening during ripening, followed by a decline associated with senescence (Figure 5c), as commonly observed in papaya storage studies.^49^ Slightly lower L* values in NS and VR treatments suggest minor effects of tag presence on light reflection. In contrast, both NS and VR tags exhibited a marked decrease in L* (≈50 to 15), indicating progressive darkening due to ethylene-driven chemical transformations (Figure 5d). Chromaticity trends were consistent, with fruit values increasing until the climacteric stage and subsequently decreasing, while tag chromaticity (30–50) confirmed gradual darkening. Additional color parameters are provided in the Supporting Information (Table S3).

Color difference (Δ*E*) analysis reinforced these findings (Table S3). Fruits showed increasing Δ*E* during storage, with CO reaching 10.6 by day 15, whereas NS and VR exhibited lower values (7.84 and 7.69), indicating moderated visual changes under active systems. In contrast, the NS tag exhibited significantly higher Δ*E* (10.5–11.2) compared to VR (4.14–4.65), reinforcing the sensor’s high sensitivity to the continuous increase in ethylene. Statistical analysis (ANOVA) showed no significant differences among fruit treatments, but a markedly higher responsiveness of NS relative to VR, consistent with its sustained ethylene adsorption capacity. These results align with reports that ethylene scavenging systems can reduce metabolic rates and delay visual ripening changes without drastically altering overall fruit color evolution.^50,51^

The fruits initially exhibited a deep green hue angle (Figures 5 and 6), transitioning to yellow between days 7 and 8, and reaching full maturity by day 11, followed by a shift toward orange (Figure 6). This progression was reflected in the RGB profiles, which shifted from dark to light green, and subsequently to a stable light orange tone. Concurrently, the NS tag displayed a distinct colorimetric transition from violet to wine and ultimately to brown, with the latter intensifying as the fruit reached the mature stage. These synchronized color shifts in the NS and CO tags provide a reliable basis for assessing papaya ripening and correlate effectively with other physicochemical parameters.

**Figure 6:**
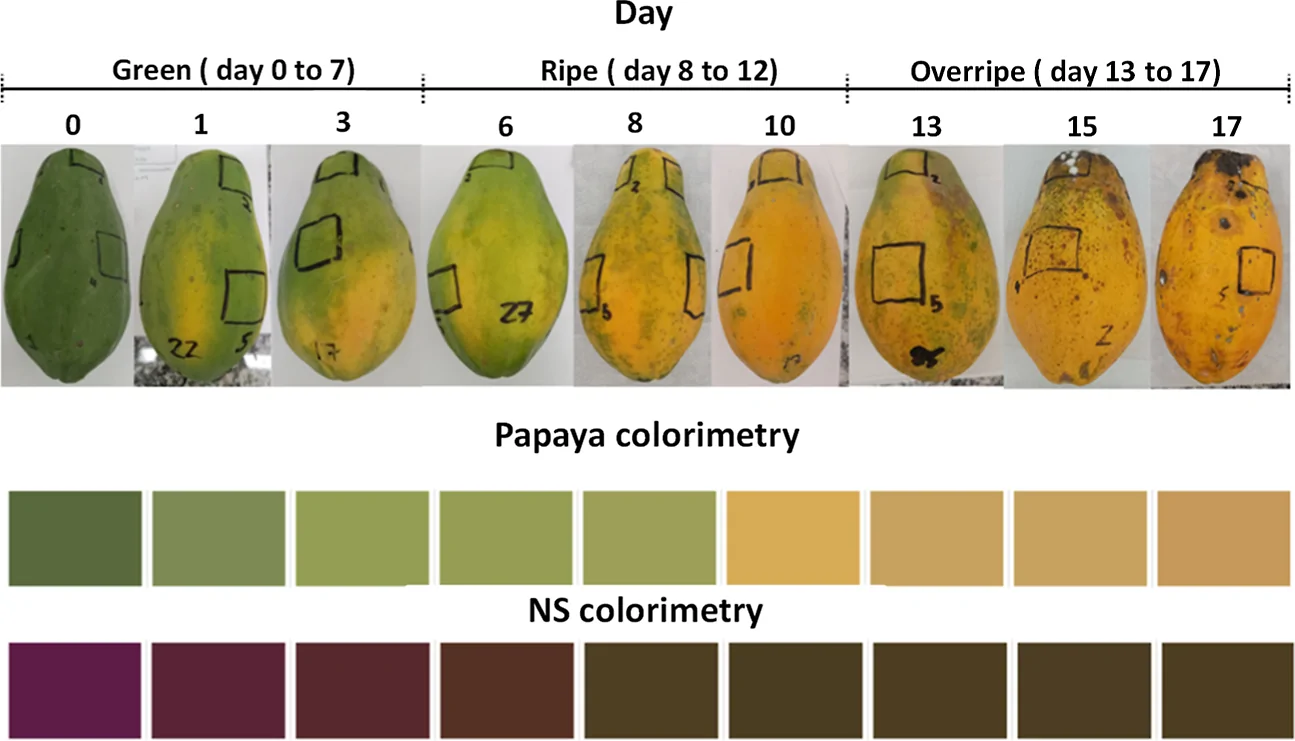
Representative color changes of papayas and permanganate-based nanosensors during postharvest ripening. Image created by the authors.

The progressive colorimetric response observed for the NS throughout storage reflects its underlying sensing mechanism. The NS operates as a single-use, irreversible colorimetric system based on the reduction of MnO_4_^–^ to MnO_2_. Consequently, its colorimetric response is irreversible, providing a cumulative record of ethylene exposure during storage. The NS exhibited a continuous color change from violet to brown, corresponding to the green-to-ripe transition of papaya during climacteric ripening (approximately 8–13 days). Each tag contained 150 mg of the nanostructured material formulated with 15 wt % KMnO_4_. Color saturation occurred only after complete depletion of the available KMnO_4_, defining the practical operational lifetime of the sensor.

### 3.3 Ethylene Release and Respiration

Although climacteric fruits release a complex mixture of volatile organic compounds (VOCs) during ripening, ethylene is the preferred biomarker because it is the primary phytohormone regulating the climacteric process and is produced before most aroma-related volatiles. Its low molecular weight (28.05 g mol^–1^) and density (≈1.15 g L^–1^), nearly identical to that of air, promote rapid diffusion throughout the package headspace, whereas denser VOCs, such as ethanol, aldehydes, and esters, accumulate later and are more dependent on local transport conditions. Consequently, potassium permanganate (KMnO_4_)-based indicators predominantly respond to ethylene, since its continuous early emission and high diffusivity make it the main reactant available for oxidation, despite the nonselective oxidizing nature of KMnO_4_. Therefore, ethylene provides the most reliable and universal indicator of fruit ripening for intelligent packaging applications.^52^ In the Supporting Information (Figure S7) presented one experiment previous of follow-up color transformation of NS from injection amount ethylene at vials controlled, showing the direct relationship with gas.

Therefore, the calibration curve (Figure 7a, inset) exhibited excellent linearity (R^2^ = 0.999), enabling accurate quantification of ethylene from chromatographic peak areas. Ethylene release was calculated (μL kg^–1^ h^–1^) using the linear equation (Area = 10.21 + 4.23 V), being V equal volume at μL, Table S5, considering sample mass and preconcentration time for each measurement. Figure 7a also presents the average ethylene release profiles for all treatments, while individual fruit curves are provided in the Supporting Information (Figure S8).

**Figure 7:**
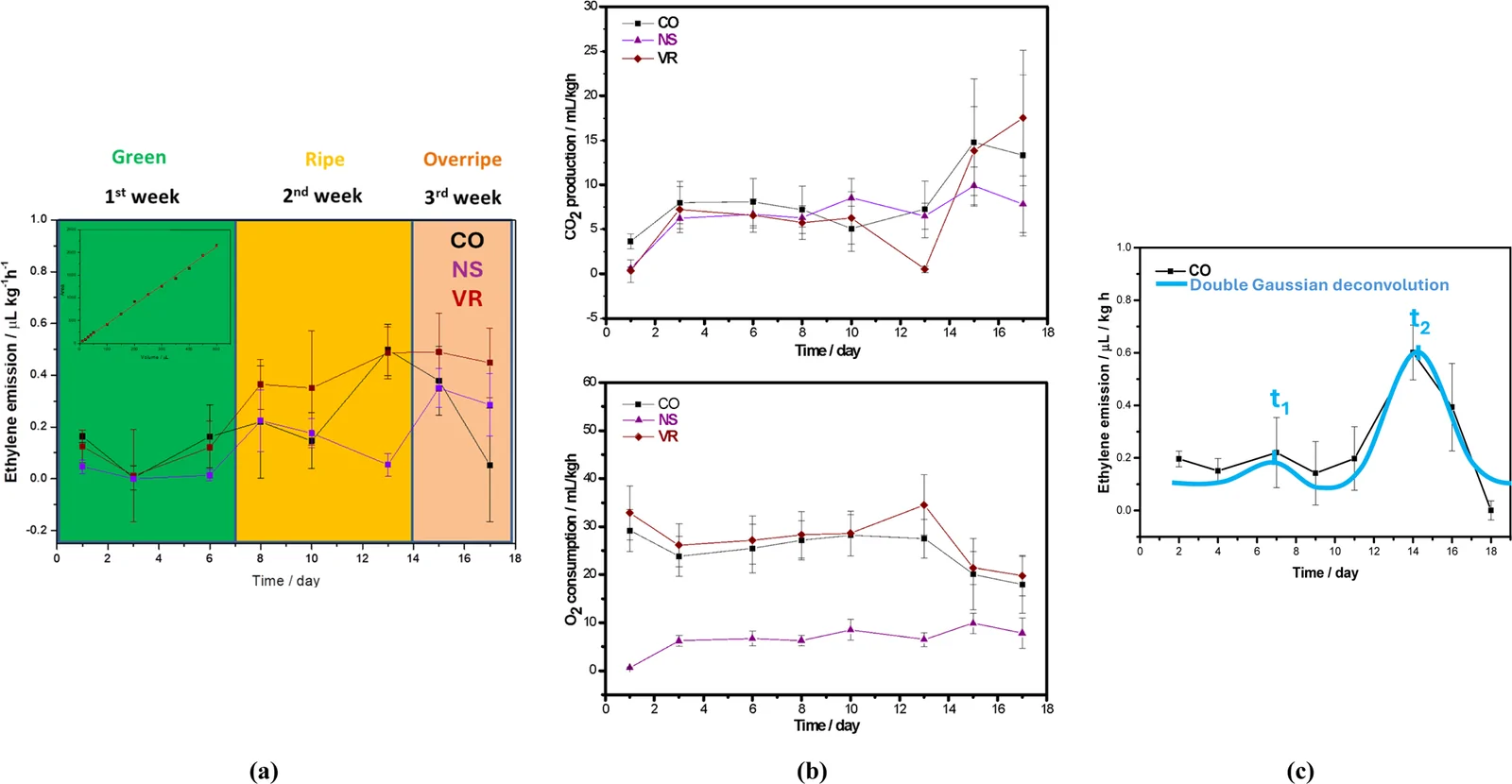
(A) Calibration curves (insert upper left) and comparison of the average ethylene release curves of the groups: CO, NS and VR. (b) Comparison of the means and standard deviations of CO_2_ production (upper) and O_2_ consumption (lower) of the CO. NS and VR groups. (c) Double Gaussian deconvolution of the ethylene release profile (CO group), highlighting peak timing parameters (t_1_ and t_2_). The bars in all graphs represent the average standard deviation, obtained from the tested packages.

The CO exhibited a typical climacteric pattern with two distinct ethylene peaks. In the initial stage, ethylene release remained below 0.2 μL kg^–1^ h^–1^, followed by a first peak around day 8 (≈0.22 μL kg^–1^ h^–1^), corresponding to the onset of ripening. A second, higher peak (∼0.50 μL kg^–1^ h^–1^) was observed at the climacteric maximum, after which ethylene production declined as the fruit entered senescence. This behavior is consistent with previous reports for papaya ripening dynamics.^49^ Daily values are provided in Supporting Information (Table S6).

In contrast, the NS treatment significantly reduced and delayed ethylene release, with the climacteric peak shifted to day 15 (vs day 13 for CO) and a lower maximum emission (0.35 vs 0.50 μL kg^–1^ h^–1^), confirming its ethylene scavenging capability. This effect is attributed to the oxidation of ethylene by permanganate-based systems, which lowers the effective ethylene concentration and delays the autocatalytic ripening process.^50^ Ethylene release under NS ranged from 0.001 to 0.35 μL kg^–1^ h^–1^, consistently below CO values.

The VR treatment showed limited and transient ethylene absorption. Although initial reductions were observed, ethylene release increased after day 8, approaching CO levels (peak ≈0.49 μL kg^–1^ h^–1^). This behavior is likely due to the moisture saturation of the aluminosilicate matrix, which preferentially adsorbs water vapor, thereby limiting further ethylene uptake. Such competitive adsorption effects have been reported for porous inorganic materials used in active packaging.^53^

Ethylene release varies across papaya species, including within the species used in this validation test, as reported in the literature.^35,44^ Additional factors, such as fruit size, harvest stage, and growing region, also influence ethylene production. Oliveira Filho et al.^35^ observed ethylene release ranging from 1.0 to 1.4 μg/kg·h over 15 days, while Bron et al.^44^ documented temperature-dependent variations, with ethylene release ranging from 0.57 to 2.0 μL/kg·h at 23 °C for different maturity stages.

### 3.4 Kinetic Behavior and General Discussion

A correlation was observed among the colorimetric response of the nanostructured sensor (NS), ethylene emission, and papaya ripening stages. The progressive increase in the hue angle of both the NS and fruit surface enabled the classification of ripening into five stages: green, transition, ripe, climacteric peak, and overripe (Table 2). While NS hue exhibited an approximately linear increase during storage, ethylene production followed a Gaussian-like climacteric profile. This relationship demonstrates that the NS provides a visual indication of ripening progression while reflecting ethylene-mediated physiological changes. The proposed model establishes a direct link between storage time, fruit maturity, ethylene evolution, and sensor color. Although developed for papaya, the approach can be extended to other climacteric fruits by adjusting the active material loading, sensor dimensions, or packaging configuration according to their specific ethylene production rates and ripening kinetics.

**Table 2:** Relation of Storage Time, Fruit Hue, Average Ethylene Emission in the CO and Degree of Maturation

day	Hue NS/deg	Hue Fruit/deg	ethylene emission CO/μL kg^–1^ h^–1^	state of ripeness
1	247.48	111.52	0.16	green
6	256.35	108.94	0.16	
8	265.49	104.88	0.22	transition
10	276.17	98.11	0.15	ripe
13	277.67	93.03	0.50	climacteric peak
15	320.13	88.60	0.38	overripe
17	326.23	83.18	0.05	

The ethylene evolution kinetics of papaya were analyzed using a production-consumption model based on the control (CO) and nanostructured sensor (NS) systems. The control data were spline-smoothed to estimate the intrinsic ethylene production profile, while the NS behavior was described by a first-order removal term. Global fitting of the experimental data yielded an apparent kinetic constant of *k* = 0.47 day^–1^, indicating effective attenuation of ethylene accumulation during the climacteric stage (8–13 days). Similar production-consumption approaches have been successfully applied to describe ethylene dynamics in postharvest systems.^54^

Although direct comparison of apparent kinetic constants among different studies is not straightforward because *k* depends on the mathematical model and experimental conditions, the obtained value is consistent with the high ethylene removal efficiency of the proposed NS. Previous studies have shown that KMnO_4_-based scavengers exhibit superior ethylene removal compared with adsorption-based materials such as zeolites because ethylene is irreversibly oxidized rather than physically adsorbed.^1^ Beyond its scavenging efficiency, the proposed NS also provides a simultaneous colorimetric indication of ethylene exposure. This dual functionality represents an important advantage over conventional scavengers, which generally operate only as passive ethylene absorbers without providing information about the ripening stage or ethylene accumulation.

Table 3 compares the proposed NS with representative ethylene scavenging and sensing systems reported in the literature. Conventional potassium permanganate-based scavengers effectively remove ethylene but generally lack real-time visual feedback. Conversely, most colorimetric indicators provide qualitative monitoring without actively modifying the storage atmosphere. The NS developed in this work combines both functionalities in a single nanostructured platform, enabling simultaneous ethylene attenuation and visual monitoring of fruit ripening. Moreover, the sensor exhibited a continuous hue transition from 247° to 326°, strongly correlated with papaya ripening, while reducing the climacteric ethylene peak by approximately 30% relative to the control system.

**Table 3:** Comparation of Art State KMnO_4_ System and Other Matrix for Sensor and Scavenger of Ethylene in the Ripe Monitoring the Fruits

system	material type	function (sensor/scavenger)	mechanism	fruit/application	visual response	ethylene removal efficiency	response time	differential
KMnO_4_ colorimetric indicator (Shin et al.)^27^	KMnO_4_ on cellulosic support	sensor + scavenger	Oxidation of C_2_H_4_ and reduction of Mn(VII)	kiwi	Yes	^a^NR	1–3 days	visual monitoring of ripening
Corrêa et al.^55^	KMnO_4_ commercial	scavenger	Oxidation of C_2_H_4_	papaya	No	reduction of the climacteric peak	8 days	control ripining
Hernando et al.^56^	Nano-KMnO_4_/nanoporous carbon	scavenger	Catalytic oxidation of C_2_H_4_	banana	No	high (qualitative)	16 days	use of confined nano-KMnO_4_
Álvarez-Hernández et al.^57^	KMnO_4_/sepiolite	scavenger	Oxidation	damascus	No	improvement in service life	^a^NR	porous mineral support
Modified zeolite^58^	Cu/Ag-zeolite	scavenger	Adsorption of ethylene	various fruits	No	moderate	hours	low cost
Photocatalytic TiO_2_ ^1^	TiO_2_	scavenger	Photocatalysis	various fruits	No	high under UV	hours	need lighting
Pd-zeolite^59^	Pd-zeolite	scavenger	Catalytic adsorption	apple, banana	No	high	hours	high adsorptive capacity
KMnO_4_/activated alumina^1^	KMnO_4_/Al_2_O_3_	scavenger	Oxidation of C_2_H_4_	banana	No	high	days	established comercial technology
KMnO_4_/vermiculite^1^	KMnO_4_/vermiculite	scavenger	Oxidation of C_2_H_4_	mango, banana	No	moderate-high	days	low cost
NS (this work)	KMnO_4_/NanoSiO_2_	sensor + scavenger	Oxidation of C_2_H_4_ with reduction MnO_4_^–^ → MnO_2_	papaya	Yes	≈30% reduction in peak climacteric	continuous monitoring for 17 days	integrates real-time visual monitoring and active ethylene removal on the same platform

a NR = not registered.

Moreover, to establish a quantitative relationship between the colorimetric response of the NS and ethylene exposure, additional calibration experiments were performed using known volumes of ethylene gas injected into the sealed test chamber. The calibration curves (Figure S6c–e) demonstrated a strong linear relationship between the hue angle and the injected ethylene volume (μL). The overall calibration exhibited an R^2^ of 0.960, while the two linear response regions showed R^2^ values of 0.990 and 0.979, respectively, indicating excellent agreement over the evaluated range. The corresponding regression equations were Hue = 255.87 + 0.196 V, Hue = 248.35 + 0.349 V, and Hue = 261.61 + 0.174 V, where V is the injected ethylene volume (μL). These results demonstrate that the NS provides a quantitative colorimetric response to ethylene exposure and can be used to estimate ethylene levels under controlled conditions. During the *in vivo* storage experiments, the sensor response was further validated by its strong correlation with ethylene production (μL kg^–1^ h^–1^) and fruit ripening progression.

The average CO_2_ production and O_2_ consumption profiles are shown in Figure 7b (upper: CO_2_; lower: O_2_). Fruit respiration followed the typical progression from the green stage through ripening to senescence.^35,44^ Clear differences in CO_2_ production were observed among treatments. CO_2_ ranged from 3.8 to 15.1 mL kg^–1^ h^–1^ for CO, 0.1 to 17.4 mL kg^–1^ h^–1^ for VR, and 0.8 to 9.9 mL kg^–1^ h^–1^ for NS. O_2_ consumption exhibited similar trends: CO decreased from 28.9 to 16.8 mL kg^–1^ h^–1^, with a peak between the green and mature stages, followed by a decline during senescence. VR showed a comparable profile (32.7 to 19.7 mL kg^–1^ h^–1^), peaking at day 13, while NS consistently exhibited lower rates (10.3 to 0.4 mL kg^–1^ h^–1^), indicating reduced respiration and delayed metabolic activity.

The reduction in respiration can be mechanistically attributed to the decreased availability of ethylene in the package headspace, resulting from its adsorption and oxidation by the NS system. As ethylene regulates climacteric respiration through autocatalytic pathways, its removal disrupts the positive feedback loop that accelerates metabolic activity, thereby delaying ripening and senescence. In contrast, the VR system showed only transient effects, likely due to competitive adsorption with water vapor, which limits its capacity to retain ethylene over time. This behavior is consistent with the physicochemical properties of aluminosilicate matrices, which preferentially bind moisture under high humidity conditions.^53^

Reported respiratory rates for papaya vary widely depending on cultivar, maturity stage, and storage conditions. For instance, Oliveira-Filho et al.^35^ reported O_2_ consumption of 35–90 μg kg^–1^ h^–1^ and CO_2_ production of 50–90 μg kg^–1^ h^–1^, while Paull et al.^45^ observed CO_2_ production between 5.0 and 45.0 mL kg^–1^ h^–1^, and Bron et al.^44^ reported values of 25–42.5 mL kg^–1^ h^–1^ depending on maturity stage. Huang et al.^46^ also demonstrated that respiration rates (7.3–33.1 mL kg^–1^ h^–1^) are strongly influenced by storage conditions. Overall, the values obtained in this study are consistent with literature ranges; however, the presence of adsorbent materials (NS and VR) contributed to reduced respiratory rates, particularly in the NS treatment.^47,48^

The system consisting of PET packaging, fruit, and adsorbent/ethylene-transforming materials reaches a dynamic equilibrium among ethylene release, CO_2_ production, and O_2_ consumption. At this stage, nonreactive gases may bind to the adsorbent through secondary interactions, reducing detectable gas volumes. VR’s layered structure can trap light gases, competing with water vapor and ethylene absorption.^60^

Although papaya was used as a representative climacteric fruit model in this study, the proposed NS is expected to be applicable to other ethylene-producing fruits, since its sensing mechanism is based on the oxidation of ethylene rather than on fruit-specific characteristics. Nevertheless, differences among fruit species, cultivars, fruit sizes, and storage temperatures may affect ethylene production rates and package headspace dynamics, thereby influencing the quantitative response of the sensor. Therefore, fruit-specific calibration models will likely be required for applications involving markedly different fruit matrices.

### 3.5 Correlation Statistic Data Fruit and Tags

Figure 7c shows the average ethylene release for the CO treatment and its deconvolution using a double Gaussian model, which accurately resolved the two emission peaks and their corresponding times (t_1_ and t_2_). Peak times were statistically evaluated by ANOVA followed by Tukey’s test to assess differences among treatments.

Despite inherent variability among individual fruits, a consistent climacteric pattern was observed across CO, NS, and VR. For CO, t_1_ ranged from 4.33 to 7.49 days and t_2_ from 11.49 to 16.46 days, with intervals (t_2_–t_1_) between 5.00 and 10.46 days. NS showed delayed ripening, with t_1_ between 7.08 and 8.37 days and t_2_ between 14.02 and 16.00 days (beyond t_2_–t_1_ from 6.51 to 8.08 days), while VR exhibited broader variability (t_1_: 2.45–9.16 days; t_2_: 8.46–15.76 days; t_2_–t_1_ from 3.92 to 10.6 days).

Ethylene emission in papaya is characterized by two peaks: the first (t_1_) marks the onset of ripening and the activation of autocatalytic ethylene production, while the second (t_2_) corresponds to the climacteric maximum and progression toward senescence, associated with processes such as pulp softening, sugar accumulation, and peel color changes.^26^ Compared to CO (t_2_ = 13.17 days), NS delayed the climacteric peak to 14.98 days, indicating extended shelf life, whereas VR showed minimal effect (t_2_ = 13.08 days), likely due to reduced ethylene selectivity and competitive adsorption with water vapor.

These results are consistent with active packaging systems based on inorganic matrices, such as zeolites, silica, clays, and alumina functionalized with oxidizing agents (e.g., KMnO_4_), which enhance ethylene adsorption and oxidation, thereby slowing ripening. Such systems have been reported to extend shelf life by 7–14 days through combined adsorption and catalytic mechanisms.^61,62^ In this context, the NS system demonstrated comparable performance, extending ripening to approximately 15 days. Statistical analysis (n = 10) confirmed a significant effect on ripening onset (t_1_, p = 0.045), while only a marginal effect was observed for the climacteric peak (t_2_, p = 0.053), and no significant differences were found for the t_2_–t_1_ interval (*p* > 0.05). These findings indicate that the NS effectively modulates ethylene dynamics, primarily by delaying the early stages of ripening.

Taken together, the results demonstrate that the proposed NS provides a multifunctional approach for ethylene management during papaya storage. Unlike previous systems that predominantly evaluate either the colorimetric response of permanganate-based materials or their ethylene-scavenging capacity, the present approach integrates the chemical removal of ethylene with a visible color change, quantitative monitoring by GC-FID, and kinetic and statistical analyses. The progressive transition from violet to brown observed in the NS was associated with ethylene exposure during fruit ripening, while the chromatographic measurements provided quantitative evidence of the temporal evolution of ethylene throughout storage. Moreover, the incorporation of the NS into a tag positioned in the package lid allowed the scavenging system to be evaluated under practical storage conditions and demonstrated its potential to delay ripening-related quality deterioration and extend the storage period of papaya. Therefore, the main contribution of this study is not only the development of a KMnO_4_/nano-SiO_2_-based ethylene scavenger, but the integration of visual indication, quantitative ethylene monitoring, kinetic interpretation, and real-package application into a single system. This integrated strategy provides a practical framework that may be adapted to other climacteric fruits after appropriate adjustment of the scavenging capacity to their ethylene production profiles.

## 4 Conclusion

An innovative nanostructured sensor (NS) was successfully developed and validated for smart packaging applications, demonstrating dual functionality as an ethylene scavenger and ripening indicator. Structural and morphological analyses (FTIR, SEM-EDS, and FEG) confirmed the successful integration of the reactive matrix into a functional tag suitable for packaging systems. The NS effectively tracked fruit ripening, as supported by statistical analysis of ethylene emission profiles, including curve deconvolution, and by its characteristic color transition driven by the reduction of permanganate (MnO_4_^–^, Mn^7+^) to MnO_2_ (Mn^4+^). This response showed strong correlation with fruit physiological changes, including color evolution, respiration, and nondestructive measurements. GC-FID analysis confirmed its superior ethylene scavenging capacity, with significantly lower emission at the climacteric peak (0.35 μL kg^–1^ h^–1^) compared to control (0.50 μL kg^–1^ h^–1^) and VR (0.49 μL kg^–1^ h^–1^). The delay of the second ethylene peak from day 13 (CO/VR) to day 15 (NS) further demonstrates shelf life extension under NS treatment. The continuous hue shift (240° to 320°) aligned with ripening progression, confirming its effectiveness as a real-time, colorimetric indicator coupled with active ethylene removal. The key novelty of this work lies in the integration of sensing and scavenging functionalities within a single nanostructured platform, enabling simultaneous monitoring and control of the ripening process. Unlike conventional systems that operate either as passive indicators or as ethylene absorbers, the NS provides a dynamic and responsive interface capable of tracking and influencing fruit physiology in real time. From an applied perspective, this technology offers a new solution for reducing postharvest losses, improving quality management, and enhancing supply chain efficiency for climacteric fruits. By coupling visual indication with active atmosphere modulation, the NS platform represents a promising advance toward next-generation smart packaging systems with direct relevance to food preservation, distribution, and sustainability.

## Supplementary Material



## Notes

The Supporting Information contains the figures and tables related to the validation of the NS using papaya through both nondestructive and destructive analyses. Figure S1 shows representative images of the tags placed inside closed chambers to evaluate color stability after 1, 15, and 30 days of storage. Figure S2 presents the FTIR spectra of the starting reagents, the NS, and the tag components, while Figure S3 compares the FTIR spectra of the NS after 1 and 30 days, demonstrating its chemical stability during storage. Figure S4 illustrates the mass variation of the NS over the storage period. Peel and pulp firmness results are presented in Figure S5. Figure S6 displays the linear regression models for the Hue-time curves obtained from the NS and the control (CO), as well as the Hue-ethylene correlation based on the additional experimental data. Figure S7 illustrates the color changes of the NS induced by different ethylene concentrations under closed-chamber conditions. Finally, Figure S8 presents the ethylene release profiles quantified by GC-FID. Tables S1 and S2 summarize the firmness measurements and disease severity index, respectively, whereas Table S3 reports the color parameters (L*, a*, and b*). Table S4 presents the ANOVA and Tukey’s post hoc statistical analyses performed for mass loss, pH, titratable acidity (TA), total soluble solids (TSS), TSS/TA ratio, and firmness. Table S5 summarizes the analytical parameters obtained from the ethylene and Hue curves regression models. Finally, Table S6 reports the individual ethylene release values determined for each fruit treatment (PDF)

## Abbreviations Used

C*: Chroma
CEASA: Araquarara Terminal Market;
CO: Control group
EDS-SEM: Energy Dispersive X Rays Scanning Electron Microscopy
EFSA: European Food Safety Authority
FDA: Food and Drug Administration
FEG-SEM: Field Emission Gum Scanning Electron Microscopy
FID: Flame Ionization Detector
FTIR: Fourier Transform Infrared Spectroscopy
GC: Gas cromatography
h°: Hue angle
IUPAC: International Uion of Pure and Applied Chemistry
L*: Luminosity
NS: Nanosensor or nanosensor group
NWF: Nonwoven fabric
PET: Polyterephthalate
RGB: Red, Green and Blue
SEM: Scanning Electron Microscopy
TSS: Titratable soluble solids
t1: time of first peak in day
t2: time of second peak in day
t2-t1: difference between peaks in day
TA: Titratable Acidity
Tag: Packaging with material
TCD: Thermal Conductivity Detector
VOCs: Volatile organic compounds
VR: Vermiculite or vermiculite group
Δ*E*: Color difference
