# Supplementary material for: Smart Nanosensor Technology for Fruit Preservation: In vivo Papaya Model with Ethylene Scavenging and Colorimetric Indication
Source: ACS Omega. 2026 Sep 21;11(39):59311–26. doi: 10.1021/acsomega.6c05286 (PMC13647914; doi:10.1021/acsomega.6c05286)
Supplement: Supplementary file 1 [file ao6c05286_si_001.pdf]

Smart nanosensor technology for fruit preservation: *In vivo* papaya model with ethylene scavenging and colorimetric indication

Ricardo dos Santos Medeiros[1*]; Ana Carolina Gava de Barros da Silveira[1]; Giovanna Maia[2]; Matheus Godoy Reimberg Amaral[2]; Ramon Peres Brexó[3]; Ednaldo José Ferreira[3]; Elaine Cristina Paris[3]; Marcos David Ferreira[3]

[1] Institute of Chemistry (IQSC), University of São Paulo (USP), São Carlos, SP, 13566-590, Brazil

[2]Department of Chemistry, Federal University of São Carlos (UFSCar), São Carlos, SP, 13565-595, Brazil

[3]Brazilian Agricultural Research Corporation, Embrapa Instrumentation, São Carlos, SP, 13561-206, Brazil

* Corresponding author:

Dr. Ricardo dos Santos Medeiros, e-mail: medeirossricardo@usp.br

Instituto de Química de São Carlos – IQSC/USP

Avenida Trabalhador São-carlense, 400 - CEP 13566-590 - São Carlos - SP - Brasil

Supporting information

Figures section

Day	Sample	Hue / °	S* = hi/hf x 100
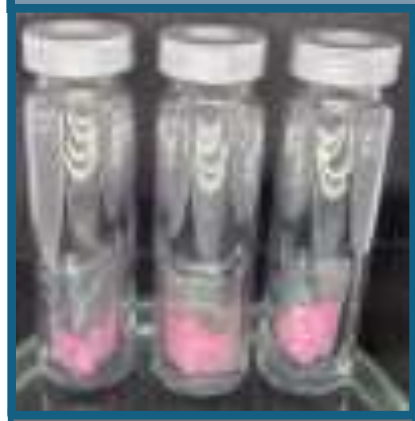	1	244.21	100.0
	15	246.51	99.1
1 15 30	30	252.52	96.7

S* equal stability; hi (initial) and hf (final) equal to hue angle value.

Figure S1. Color (hue angle) and stability value data about change color of NS at chamber closed control.

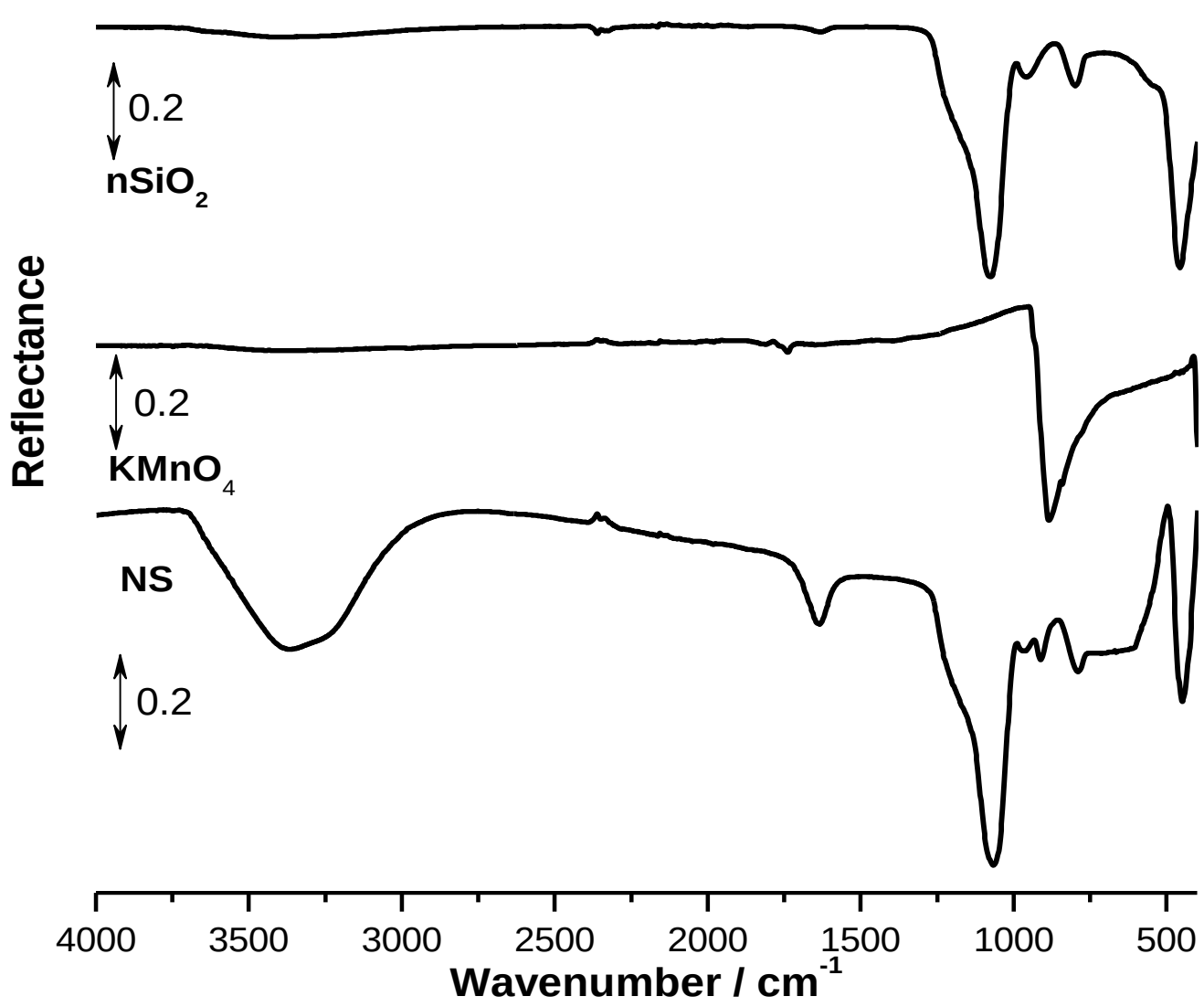

(a)

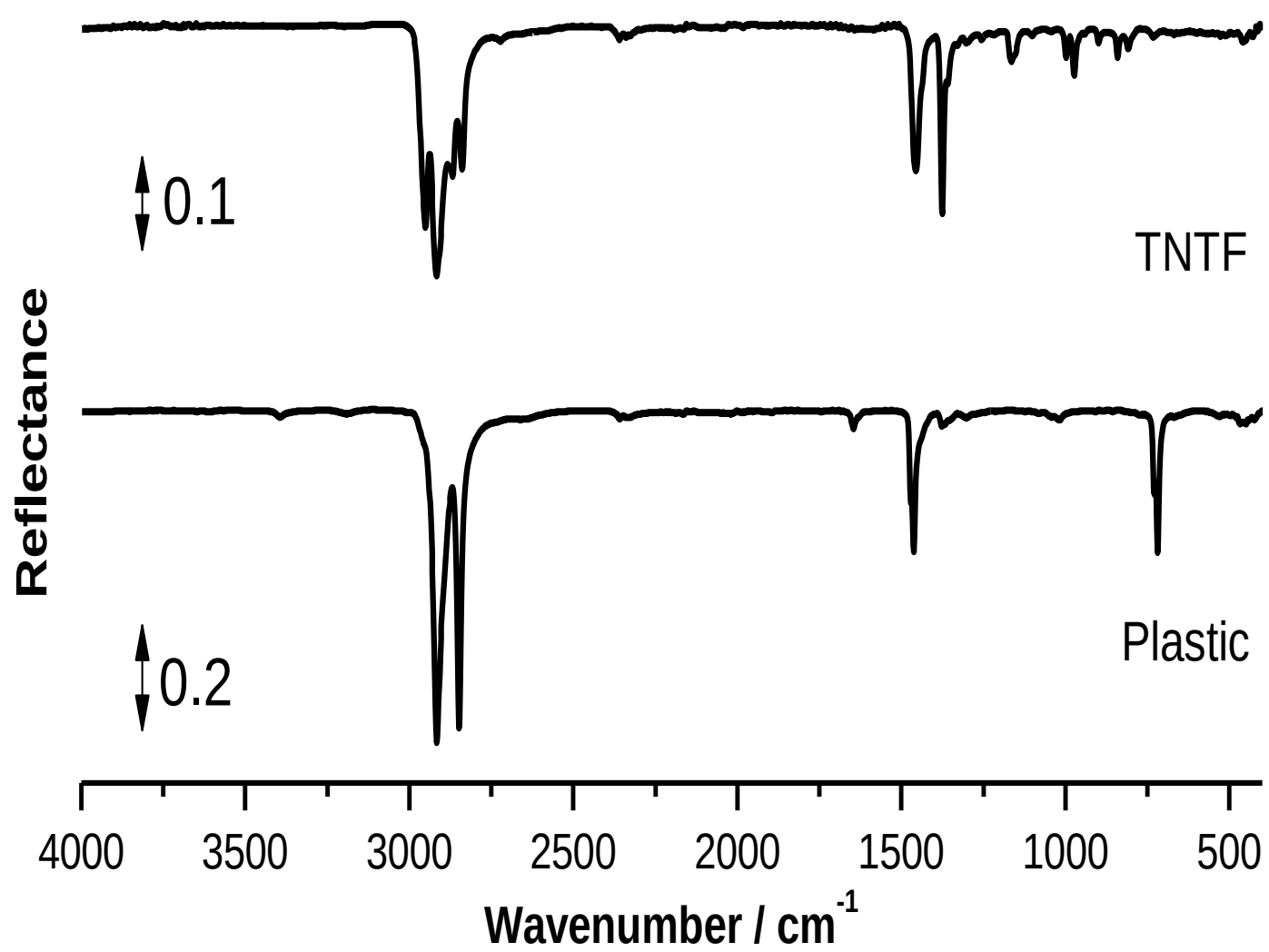

(b)

Figure S2. (a) FTIR comparison of the nanosensor components; (b) FTIR spectra of the tag components

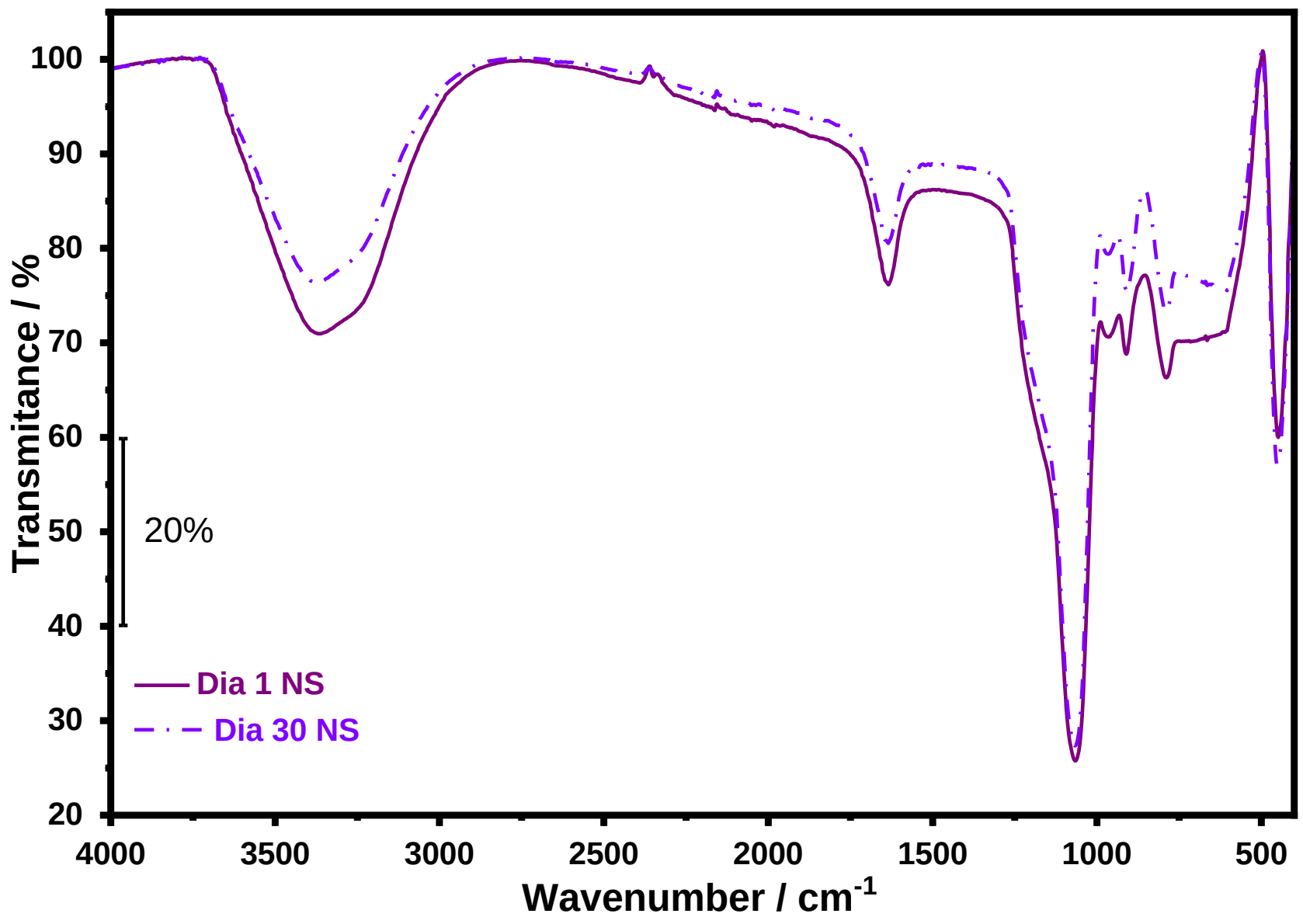

Figure S3. FTIR spectra of NS relates to 1 (after preparation) and after 30 days storage of syntheses (chemical stability).

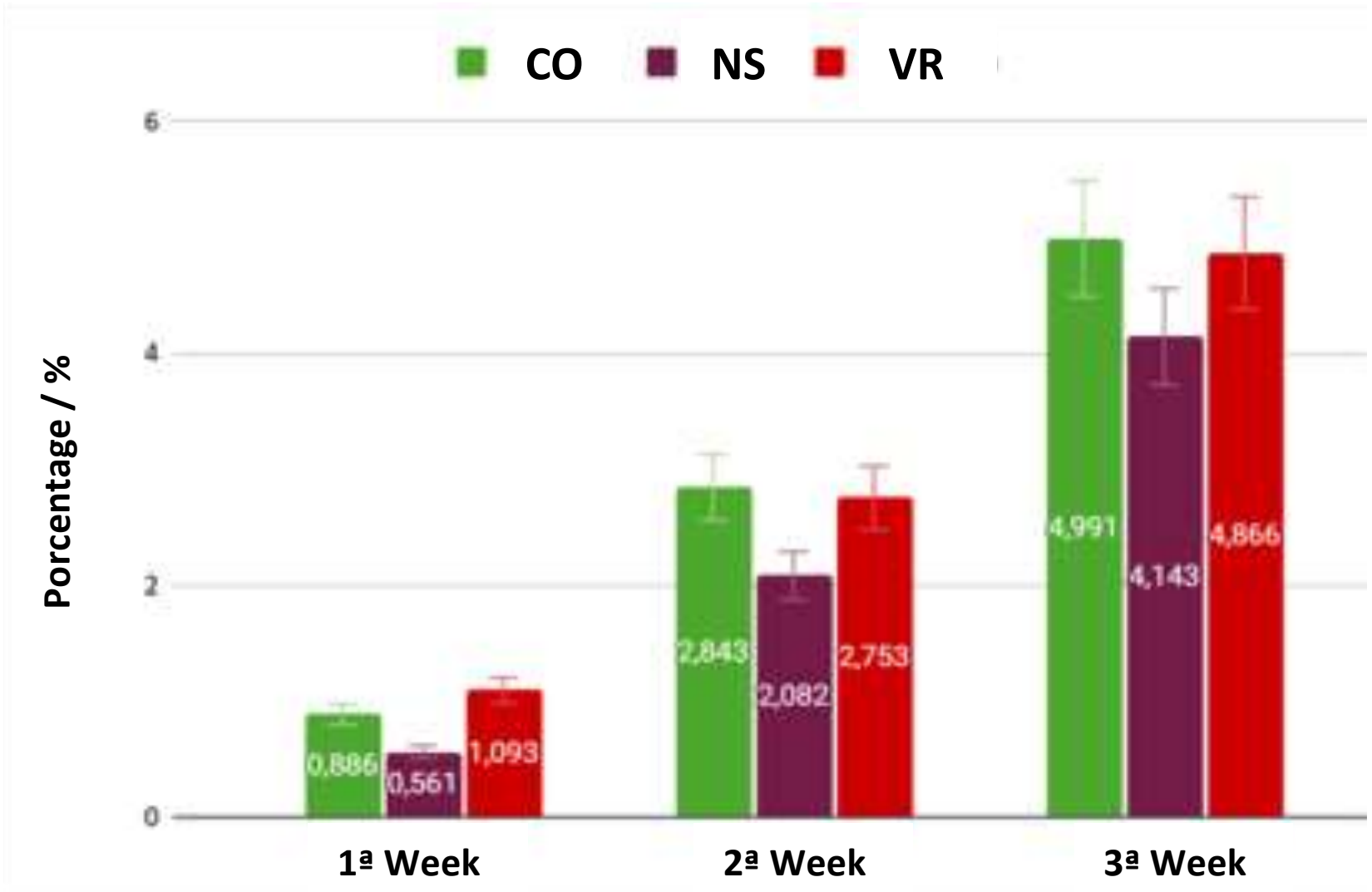

Figure S4. Mass loss variation of the general test.

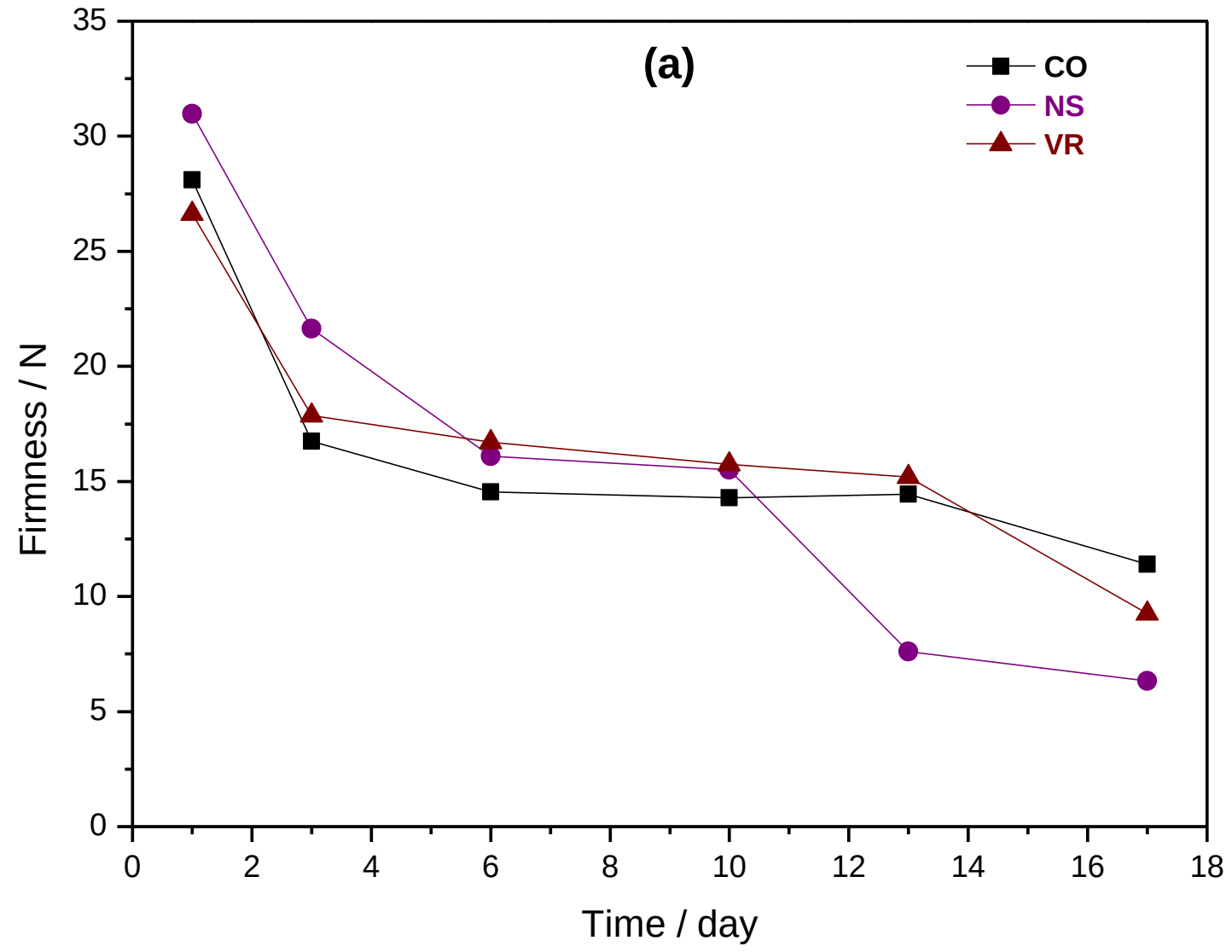

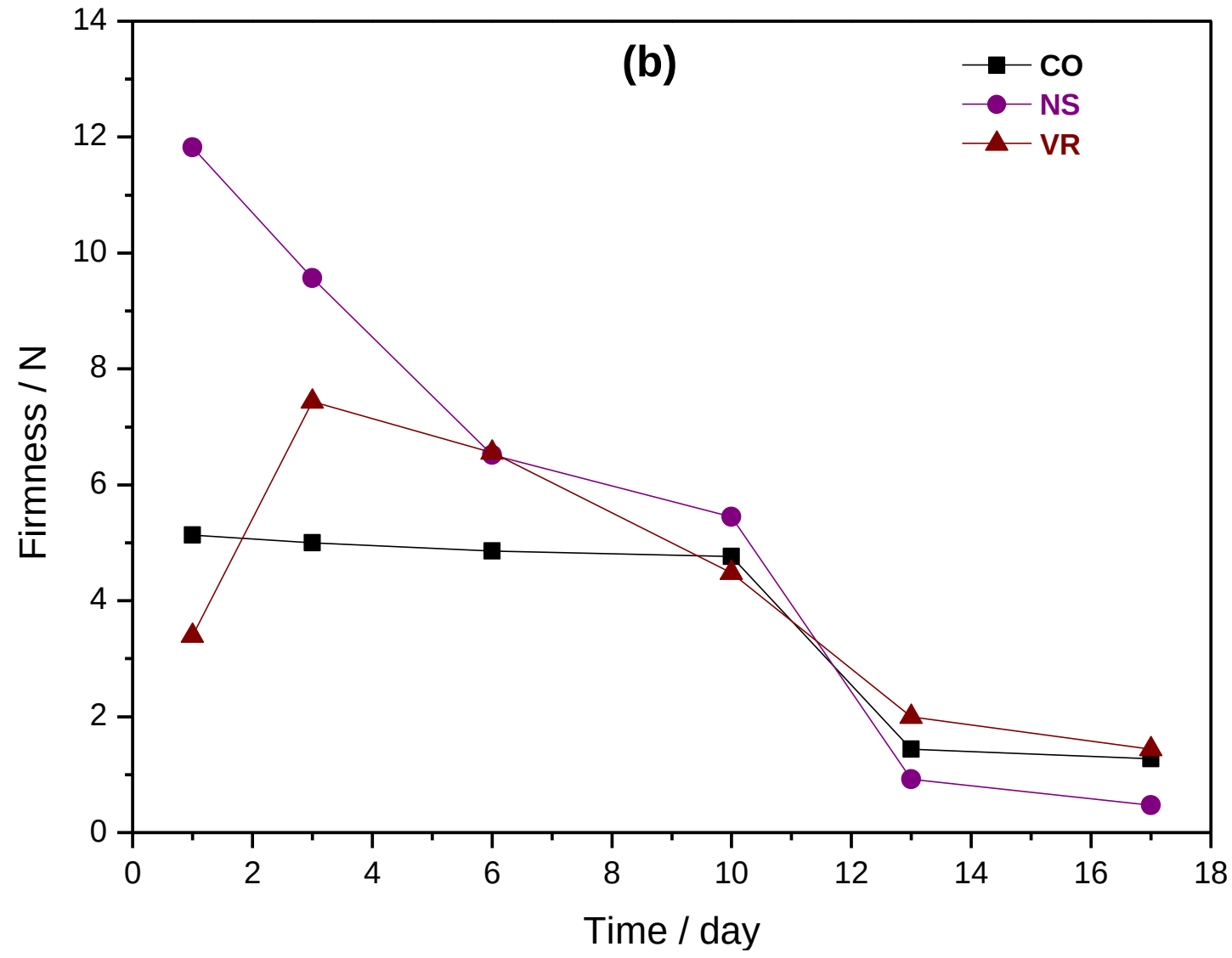

Figure S5. Graphs of firmness in Newtons versus time in days for the study groups. (a) peel and (b) pulp.

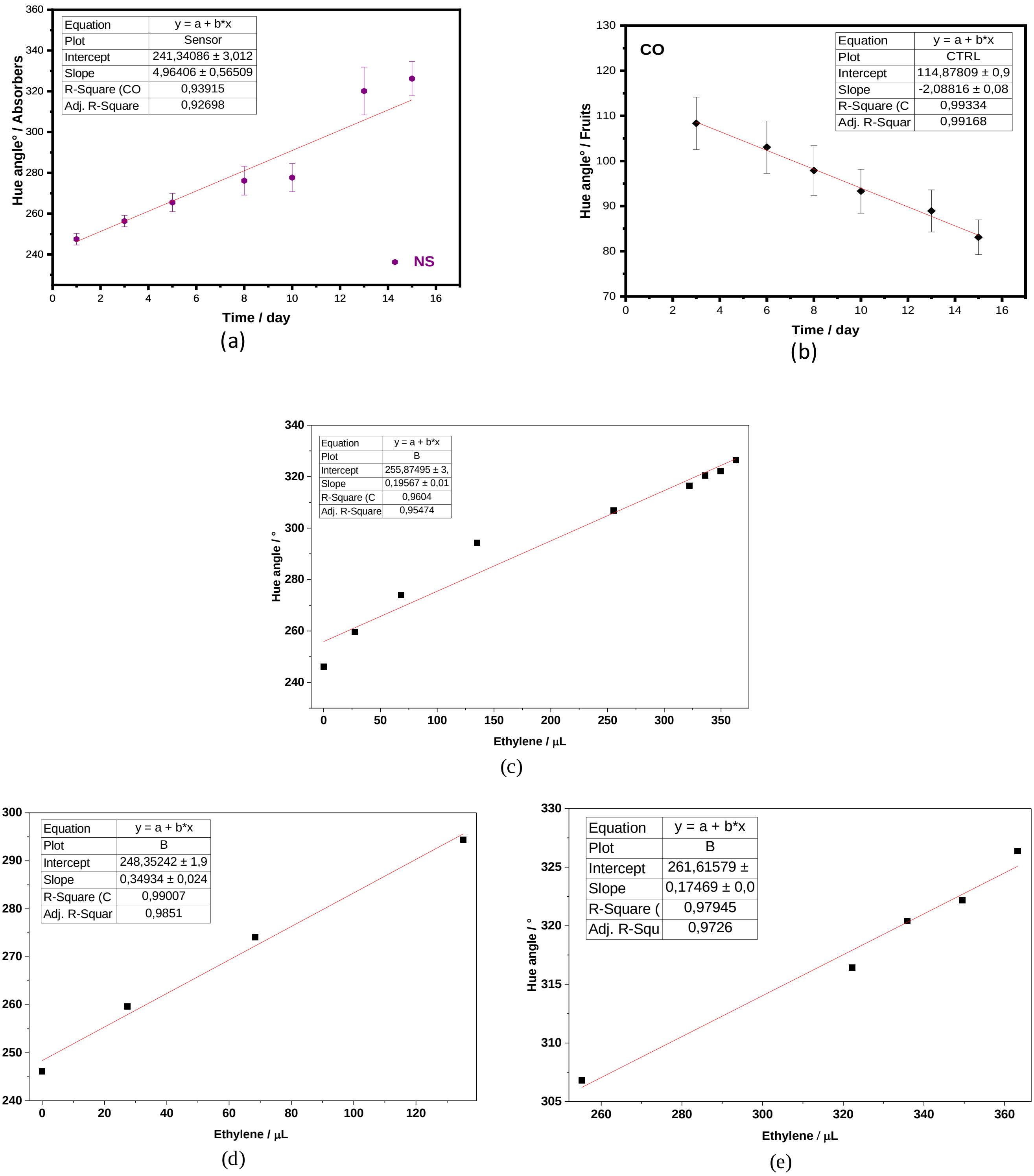

Figure S6. Regression linear applied for (a) hue angle x time for NS; (b) hue angle x time for treatment CO; (c) relationship of NS hue angle X ethylene experiment additional for evaluating the change color of system(integral curve); (d) Splite the integral curve (first region) and (e) (second region).

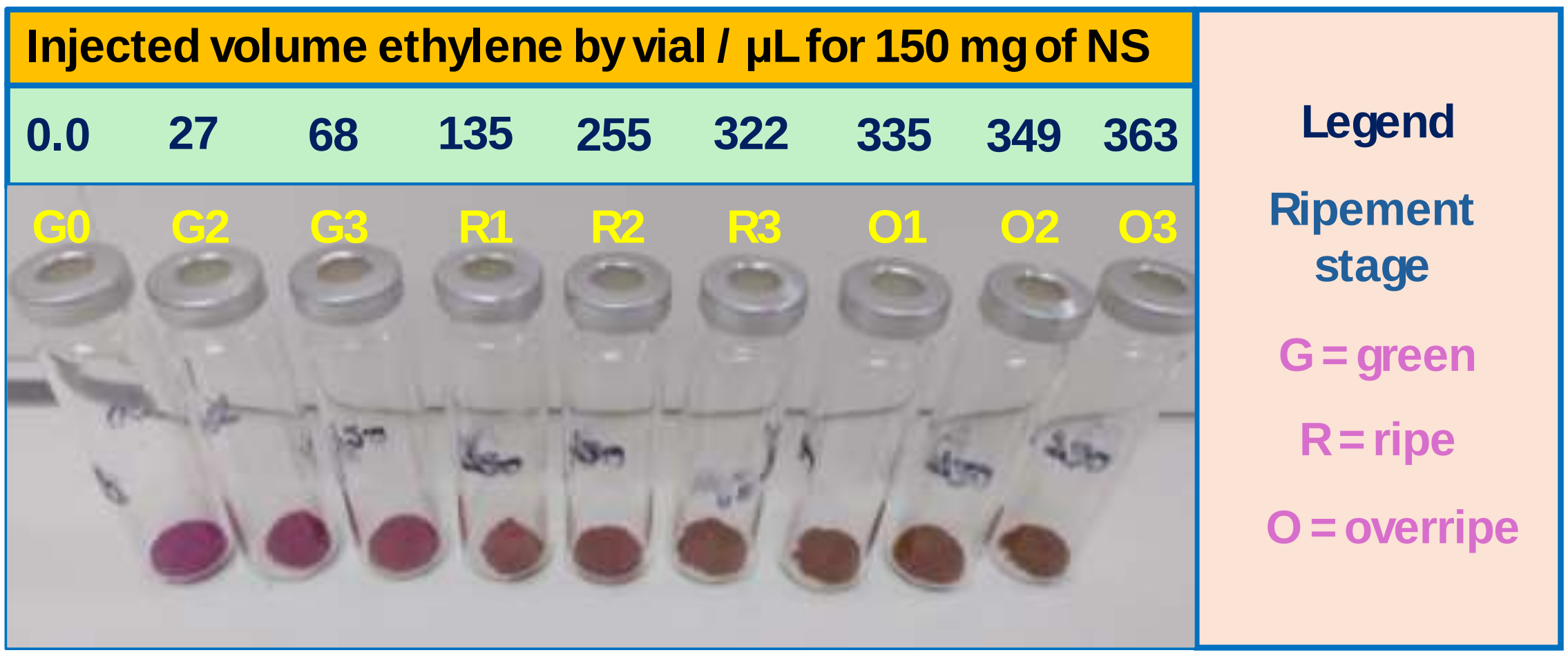

Figure S7. Imagens of follow-up of experiment with ethylene fraction for test color transformation of NS.

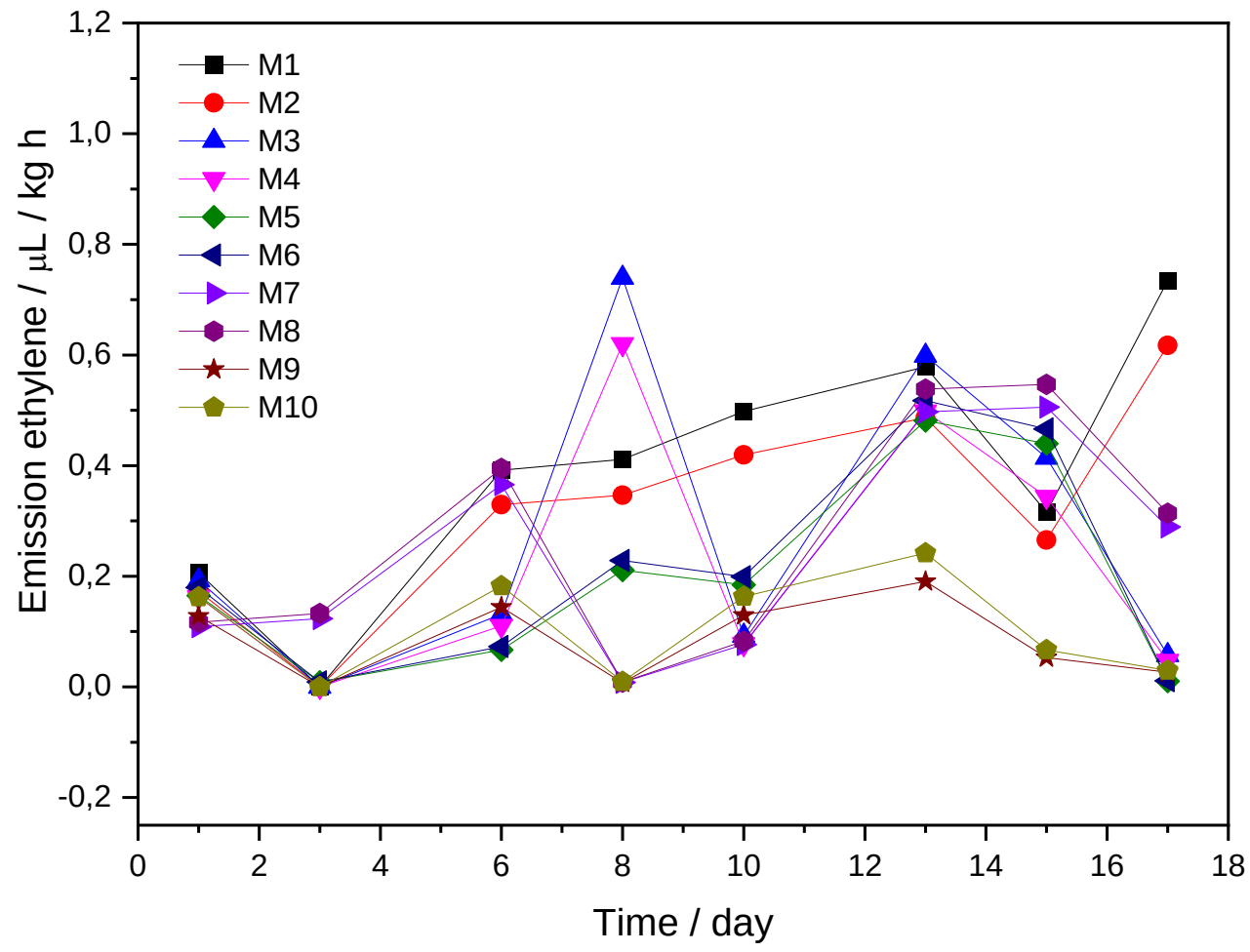

(a)

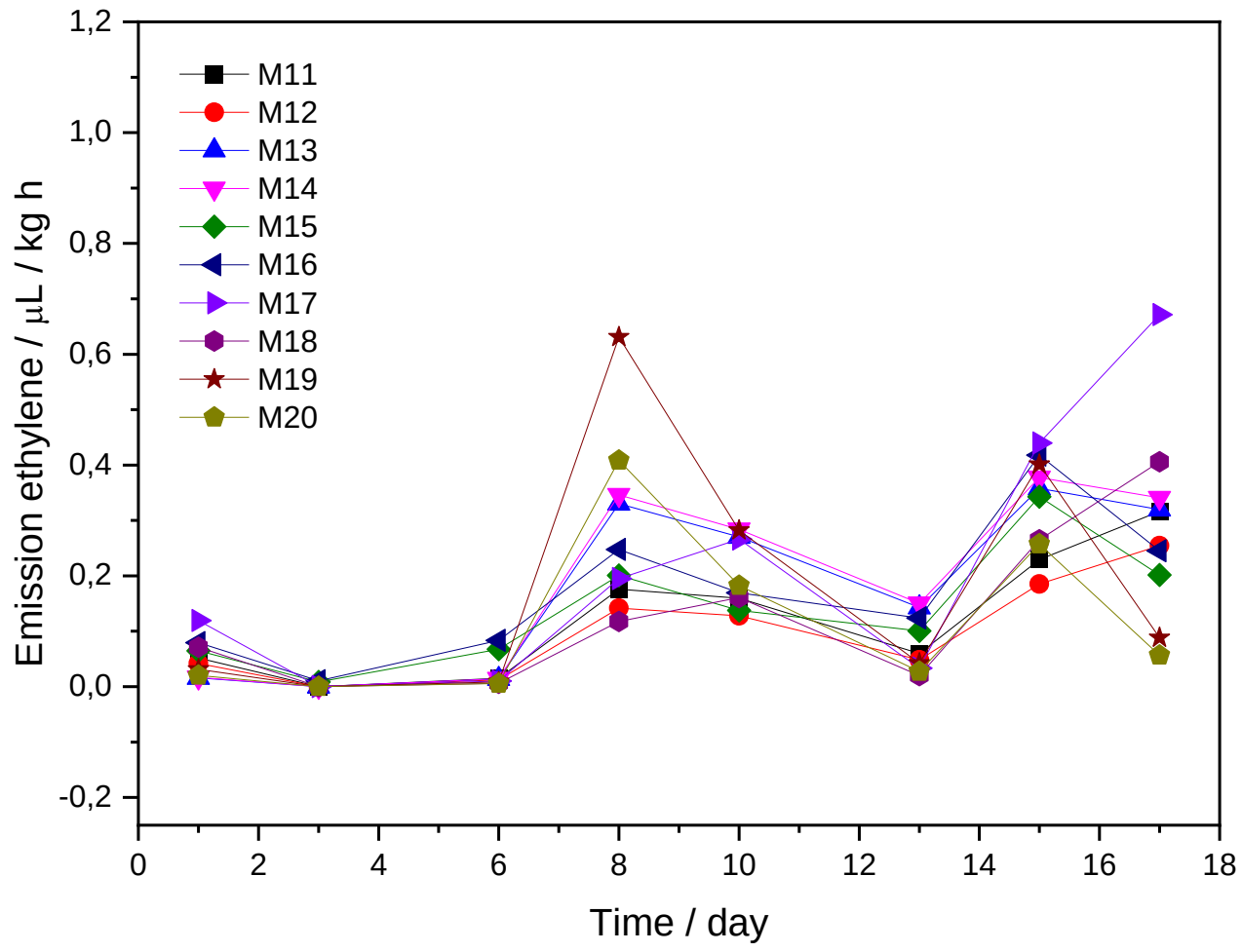

(b)

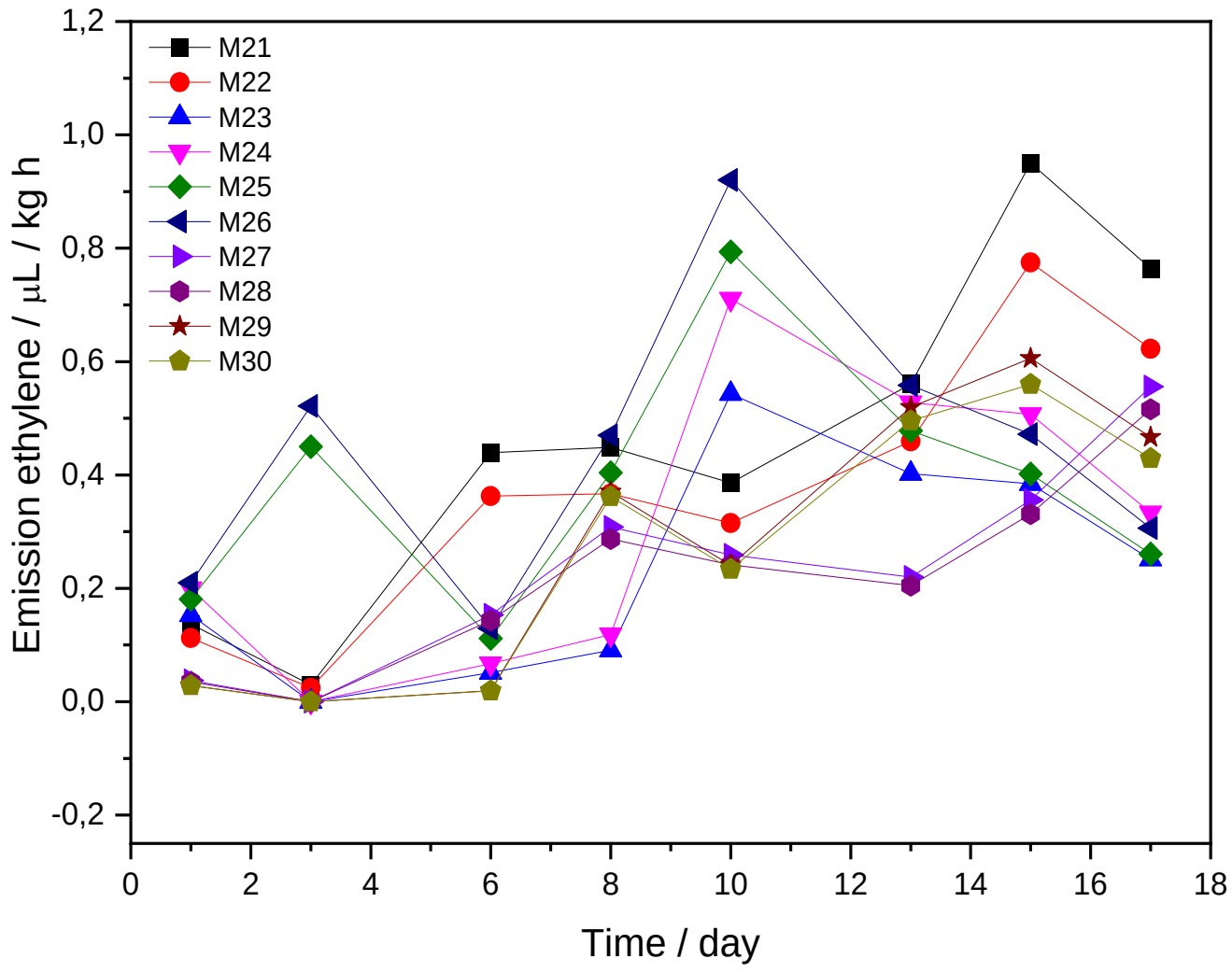

(c)

Figure S8. Comparison of ethylene release curves per kilogram hour for groups (a) CO; (b) NS (c) VR.

Table section

Table S1. Results of firmness (N) of papaya peel and pulp

Treatments	Time stored / day																							
	1				3				6				10				13				15			
	Peel	SD*	Pulp	SD*	Peel	SD*	Pulp	SD*	Peel	SD*	Pulp	SD*	Peel	SD*	Pulp	SD*	Peel	SD*	Pulp	SD*	Peel	SD*	Pulp	SD*
CO	28.10	1.22	5.12	0.72	16.74	4.9	5.00	3.16	13.58	1.09	4.85	1.50	14.28	4.36	4.76	2.39	14.43	6.46	1.44	0.19	11.39	3.75	1.27	0.33
NS	30.97	2.8	11.82	7.54	21.64	4.75	9.56	4.23	16.09	5.17	4.39	2.16	15.51	10.42	5.44	1.16	7.60	2.41	0.92	0.15	6.31	2.85	0.46	0.22
VR	26.63	7.43	3.39	0.98	17.86	2.66	7.43	1.08	16.70	1.06	3.11	1.99	15.74	5.22	4.47	2.7	15.19	2.73	1.04	0.6	9.25	5.79	1.55	0.27

*SD = standard deviation.

Table S2. Description of the fruit severity index for the CO. NS. and VR groups

Time	CO										NS										VR									
**Dia**	**M1**	**M2**	**M3**	**M4**	**M5**	**M6**	**M7**	**M8**	**M9**	**M10**	**M11**	**M12**	**M13**	**M14**	**M15**	**M16**	**M17**	**M18**	**M19**	**M20**	**M21**	**M22**	**M23**	**M24**	**M25**	**M26**	**M27**	**M28**	**M29**	**M30**
**0**	0	0	0	0	0	0	0	0	0	0	0	0	0	0	0	0	0	0	0	0	0	0	0	0	0	0	0	0	0	0
**2**	0	0	0	0	0	0	0	0	0	0	0	0	1	0	0	1	0	0	0	0	0	0	1	0	0	0	0	0	0	0
**4**	1	0	0	1	0	1	0	1	0		0	0	1	0	1	1	0	0	0	1	0	1	1	0	1	1	0	1	1	0
**7**	1	0	0	1	1	1	0	1			0	1	1	1	1	1	0	0	1	1	1	1	1	0	1	1	0	1	1	0
**11**	1	1	1	1	1		2	1	2	0	1	1		1	1	1	1	1	1	2	1		2	1	1	1	1	1		
**14**	1	1	1	2	1	1	2	1	3	2	1	1	1	1	1	1	1		1	2			2	1	2	1	1	1	2	1
**16**	1	1	1	3			3	1	4	3	1	1	3	1	2	2	1	1			2	1	5	1	3	1	1	1	2	
**18**	1	2	2	4			4		5	4	1	1	4	1	2	3	2		2	3			5	2	4	2	2	2	3	4

Caption

0	no deterioration
1	1 to 20 % of deterioration
2	21 to 40 % of deterioration
3	41 to 60 % of deterioration
4	61 to 80 % of deterioration
5	81 to 100 % of deterioration
	Without measure

Table S3. Color parameters L*. C*. h°. and ΔE* of papayas and tag stored for 15 days. at 16°C and 50% RH

Treatments	Time / day																										
	1			3				5				8				10				13				15			
	L*	C*	(h°)	L*	C*	(h°)	ΔE*	L*	C*	(h°)	ΔE*	L*	C*	(h°)	ΔE*	L*	C*	(h°)	ΔE*	L*	C*	(h°)	ΔE*	L*	C*	(h°)	ΔE*
CO - fruit	-	-	-	57.88 ± 5.99	45.36 ± 6.61	108.35 ± 5.80	-	61.18 ± 6.08	49.16 ± 7.31	103.07 ± 5.82	5.53 ± 2.70	61.97 ± 4.77	52.21 ± 5.03	97.89 ± 5.50	8.07 ± 2.94	62.92 ± 4.03	56.68 ± 4.70	93.31 ± 4.87	6.40 ± 1.81	63.05 ± 4.05	57.23 ± 4.95	88.94 ± 4.65	4.87 ± 1.74	58.32 ± 5.50	53.46 ± 5.60	83.09 ± 3.84	10.62 ± 4.63
NS - fruit	55.74 ± 6.14	40.89 ± 7.27	111.52 ± 5.84	57.03 ± 4.20	44.96 ± 5.14	108.95 ± 5.72	10.13 ± 6.69	58.29 ± 4.22	47.23 ± 5.35	104.88 ± 6.08	4.01 ± 1.52	61.21 ± 4.38	50.46 ± 4.95	98.11 ± 5.95	7.64 ± 2.58	62.29 ± 3.82	55.57 ± 4.83	93.04 ± 5.55	7.32 ± 2.15	62.64 ± 3.95	58.02 ± 5.56	88.60 ± 5.15	6.15 ± 1.49	57.78 ± 4.36	53.36 ± 6.33	83.19 ± 4.40	7.84 ± 3.63
VR. - fruit	51.79 ± 4.83	37.68 ± 5.61	115.37 ± 4.30	53.13 ± 5.17	40.90 ± 6.53	112.90 ± 4.61	4.87 ± 4.45	54.81 ± 6.28	43.90 ± 7.75	108.75 ±4.95	4.91 ± 3.22	58.98 ± 5.02	49.38 ± 6.08	101.41 ± 5.65	7.38 ± 2.75	60.02 ± 4.41	52.74 ± 5.90	96.74 ± 5.60	8.86 ± 3.26	61.57 ± 4.47	55.61 ± 5.82	91.83 ± 4.88	5.74 ± 1.29	59.41 ± 4.84	54.48 ± 5.73	86.72 ± 3.94	7.69 ± 3.48
TAG-NS	48.83 ± 8.27	27.21 ± 4.68	247.49 ± 2.87	34.54 ± 5.57	30.65 ± 2.77	256.36 ± 2.78	15.76 ± 3.22	24.35 ± 4.77	28.57 ± 3.89	265.50 ± 4.49	12.21 ± 6.06	20.90 ± 4.31	23.83 ± 4.09	276.17 ± 7.06	10.58 ± 4.87	24.88 ± 5.18	23.96 ± 3.46	277.68 ± 6.89	9.90 ± 3.65	19.67 ± 5.40	10.91 ± 3.63	320.14 ± 11.71	18.18 ± 4.65	16.27 ± 7.20	8.45 ± 4.40	326.23 ± 8.42	11.23 ± 5.80
TAG-VR	49.52 ± 7.19	17.07 ± 2.19	264.39 ± 2.39	15.35 ± 1.81	16.22 ± 1.82	266.49 ± 1.41	25.38 ± 5.23	25.17 ± 3.55	16.81 ± 1.96	267.07 ± 1.61	3.22 ± 0.67	20.83 ± 3.18	18.61 ±2.36	267.14 ± 1.14	4.14 ± 1.79	19.52 ± 5.66	13.36 ± 1.58	267.63 ± 3.18	9.50 ± 2.06	19.26 ± 3.20	5.80 ± 1.62	268.88 ± 2.13	12.55 ± 5.76	15.89 ± 5.53	4.66 ± 1.88	269.36 ± 1.91	4.65 ± 1.87

Table S4. Results of ANOVA (one factor) non-destructive and destructive analysis

Mass

Week 1

ANOVA

Source of variation	*SQ*	*gl*	*MQ*	*F*	*p-value*	*Critical F*
Between groups	1.89228	2	0.94614	4.459226	0.017533	3.219942
Within the groups	8.911384	42	0.212176			
Total	10.80366	44				

Week 2

ANOVA

Source of variation	*SQ*	*gl*	*MQ*	*F*	*p-value*	*Critical F*
Between groups	4.250907	2	2.125453	2.345222	0.108255	3.219942
Within the groups	38.06422	42	0.906291			
Total	42.31512	44				

Week 3

ANOVA

Source of variation	*SQ*	*gl*	*MQ*	*F*	*p-value*	*Critical F*
Between groups	0.504815	2	0.252408	0.055222	0.946344	3.219942
Within the groups	191.973	42	4.570786			
Total	192.4778	44				

pH and Titratable acidity (TA)

pH
Day 1

ANOVA

Source of variation	*SQ*	*gl*	*MQ*	*F*	*p-value*	*Critical F*
Between groups	0.051409	2	0.025705	2.753577	0.116673	4.256495
Within the groups	0.084015	9	0.009335			
Total	0.135424	11				

Day 3

ANOVA

Source of variation	*SQ*	*gl*	*MQ*	*F*	*p-value*	*Critical F*
Between groups	0.006585	2	0.003293	0.345332	0.716969	4.256495
Within the groups	0.085811	9	0.009535			
Total	0.092396	11				

Day 6

ANOVA

Source of variation	*SQ*	*gl*	*MQ*	*F*	*p-value*	*Critical F*
Between groups	0.174154	2	0.087077	5.005962	0.034552	4.256495
Within the groups	0.156552	9	0.017395			
Total	0.330706	11				

Day 10

ANOVA

Source of variation	*SQ*	*gl*	*MQ*	*F*	*p-value*	*Critical F*
Between groups	0.098822	2	0.049411	2.486789	0.138104	4.256495
Within the groups	0.178825	9	0.019869			
Total	0.277647	11				

Day 13

ANOVA

Source of variation	*SQ*	*gl*	*MQ*	*F*	*p-value*	*Critical F*
Between groups	0.081867	2	0.040933	1.121461	0.367396	4.256495
Within the groups	0.3285	9	0.0365			
Total	0.410367	11				

Titratable acidity (TA)

Day 1

ANOVA

Source of variation	*SQ*	*gl*	*MQ*	*F*	*p-value*	*Critical F*
Between groups	0.002704	2	0.001352	5.187792	0.031728	4.256495
Within the groups	0.002346	9	0.000261			
Total	0.00505	11				

Day 3

ANOVA

Source of variation	*SQ*	*gl*	*MQ*	*F*	*p-value*	*Critical F*
Between groups	4.83E-06	2	2.42E-06	0.016049	0.984107	4.256495
Within the groups	0.001356	9	0.000151			
Total	0.001361	11				

Day 6

ANOVA

Source of variation	*SQ*	*gl*	*MQ*	*F*	*p-value*	*Critical F*
Between groups	0.001058	2	0.000529	5.171838	0.031964	4.256495
Within the groups	0.000921	9	0.000102			
Total	0.001979	11				

Day 10

ANOVA

Source of variation	*SQ*	*gl*	*MQ*	*F*	*p-value*	*Critical F*
Between groups	0.000442	2	0.000221	0.257013	0.778847	4.256495
Within the groups	0.007733	9	0.000859			
Total	0.008174	11				

Day 13

ANOVA

Source of variation	*SQ*	*gl*	*MQ*	*F*	*p-value*	*Critical F*
Between groups	0.000426	2	0.000213	1.125171	0.366307	4.256495
Within the groups	0.001703	9	0.000189			
Total	0.002129	11				

Day 17

ANOVA

Source of variation	*SQ*	*gl*	*MQ*	*F*	*p-value*	*Critical F*
Between groups	0.000748	2	0.000374	0.628923	0.555058	4.256495
Within the groups	0.005355	9	0.000595			
Total	0.006104	11				

Soluble solid titratable (TSS) and Ratio

Soluble solid titratable (TSS)

Day 1

ANOVA

Source of variation	*SQ*	*gl*	*MQ*	*F*	*p-value*	*Critical F*
Between groups	16.21892	2	8.10946	0.95426	0.420867	4.256495
Within the groups	76.48349	9	8.498165			
Total	92.70241	11				

Day 3

ANOVA

Source of variation	*SQ*	*gl*	*MQ*	*F*	*p-value*	*Critical F*
Between groups	2.902517	2	1.451258	0.597254	0.570746	4.256495
Within the groups	21.86898	9	2.429886			
Total	24.77149	11				

Day 6

ANOVA

Source of variation	*SQ*	*gl*	*MQ*	*F*	*p-value*	*Critical F*
Between groups	2.738101	2	1.369051	0.792092	0.482095	4.256495
Within the groups	15.55558	9	1.728398			
Total	18.29368	11				

Day 10

ANOVA

Source of variation	*SQ*	*gl*	*MQ*	*F*	*p-value*	*Critical F*
Between groups	3.126163	2	1.563081	0.485576	0.630576	4.256495
Within the groups	28.97125	9	3.219027			
Total	32.09741	11				

Day 13

ANOVA

Source of variation	*SQ*	*gl*	*MQ*	*F*	*p-value*	*Critical F*
Between groups	9.050717	2	4.525358	1.527657	0.268403	4.256495
Within the groups	26.66058	9	2.962286			
Total	35.71129	11				

Ratio

Day 1

ANOVA

Source of variation	*SQ*	*gl*	*MQ*	*F*	*p-value*	*Critical F*
Between groups	13500.11	2	6750.055	1.031053	0.395204	4.256495
Within the groups	58920.83	9	6546.759			
Total	72420.94	11				

Day 3

ANOVA

Source of variation	*SQ*	*gl*	*MQ*	*F*	*p-value*	*Critical F*
Between groups	1242.054	2	621.027	0.479534	0.640915	5.143253
Within the groups	7770.376	6	1295.063			
Total	9012.43	8				

Day 6

ANOVA

Source of variation	*SQ*	*gl*	*MQ*	*F*	*p-value*	*Critical F*
Between groups	9325.481	2	4662.74	3.518726	0.074299	4.256495
Within the groups	11926.1	9	1325.122			
Total	21251.58	11				

Day 10

ANOVA

Source of variation	*SQ*	*gl*	*MQ*	*F*	*p-value*	*Critical F*
Between groups	2016.774	2	1008.387	0.259163	0.777264	4.256495
Within the groups	35018.37	9	3890.93			
Total	37035.15	11				

Day 13

ANOVA

Source of variation	*SQ*	*gl*	*MQ*	*F*	*p-value*	*Critical F*
Between groups	8111.66	2	4055.83	2.340162	0.151935	4.256495
Within the groups	15598.27	9	1733.141			
Total	23709.93	11				

Day 17

ANOVA

Source of variation	*SQ*	*gl*	*MQ*	*F*	*p-value*	*Critical F*
Between groups	9486.756	2	4743.378	1.918019	0.202373	4.256495
Within the groups	22257.54	9	2473.06			
Total	31744.3	11				

Firmness

Day 1

ANOVA

Source of variation	*SQ*	*gl*	*MQ*	*F*	*p-value*	*Critical F*
Between groups	175.5182	2	87.7591	3.204236	0.088956	4.256495
Within the groups	246.4961	9	27.38846			
Total	422.0143	11				

Day 3

ANOVA

Source of variation	*SQ*	*gl*	*MQ*	*F*	*p-value*	*Critical F*
Between groups	52.56162	2	26.28081	1.86887	0.209496	4.256495
Within the groups	126.5617	9	14.06241			
Total	179.1233	11				

Day 6

ANOVA

Source of variation	*SQ*	*gl*	*MQ*	*F*	*p-value*	*Critical F*
Between groups	6.509113	2	3.254556	0.669235	0.535844	4.256495
Within the groups	43.7679	9	4.8631			
Total	50.27701	11				

Day 10

ANOVA

Source of variation	*SQ*	*gl*	*MQ*	*F*	*p-value*	*Critical F*
Between groups	1.971157	2	0.985579	0.153672	0.859756	4.256495
Within the groups	57.72178	9	6.413531			
Total	59.69294	11				

Day 13

ANOVA

Source of variation	*SQ*	*gl*	*MQ*	*F*	*p-value*	*Critical F*
Between groups	0.208561	2	0.104281	0.516765	0.613125	4.256495
Within the groups	1.816157	9	0.201795			
Total	2.024718	11				

Day 17

ANOVA

Source of variation	*SQ*	*gl*	*MQ*	*F*	*p-value*	*Critical F*
Between groups	3.340869	2	1.670434	6.505792	0.017871	4.256495
Within the groups	2.31085	9	0.256761			
Total	5.651719	11				

Table S5. Resume of analytical parameters for the Ethylene and Hue curves discussion in the manuscript

NS TAG	Regression equation	Intercept	Sensitivity	Linear region	Correlation coeffient, R^2	*LOD	**LOQ	t_{50}^a and t_{90}^b
Ethylene curve (Fig.7[a]-insert)	y = 10.21 + 4.23x	10.21 area	4.23 area μL^{-1}	10-500 µL	0.999	0.023 µL	0.072 µL	-
Hue angle curve (Fig.S6a NS fitted)	y = 241.34 + 4.96x	241.34 hue°	4.96 ° day^{-1}	247.5-326.2°	0.939	0.375 hue°	1.138 hue°	10.1; 15.5 days

* LOD (limit of detection) was calculated using the expression: LOD = (3.3.σ)/s; being σ = standard deviation of the white; s = curve slope;

** LOQ (limit of quantification) was calculated by expression: LOQ = (10. σ)/s; being σ = standard deviation of the white; s = curve slope;

a t_{50} is the time to reach 50% of change of color on the NS;

b t_{90} is the time to reach 90% of change of color on the NS;

Table S6. Ethylene release parameters for analysis of the CO. NS. and VR treatments

Treatments	Time / day	Ethylene emission/ µL /kg h											
		Samples											
		**M1**	**M2**	**M3**	**M4**	**M5**	**M6**	**M7**	**M8**	**M9**	**M10**	**Avarage**	**SD**
	1	0.21	0.17	0.19	0.16	0.16	0.18	0.11	0.12	0.13	0.16	0.16	0.02
	3	0	0	0	0	0.01	0.01	0.12	0.13	0	0	0.004	0.04
	6	0.39	0.33	0.13	0.11	0.07	0.07	0.36	0.39	0.14	0.18	0.16	0.12
**CO***	8	0.41	0.35	0.74	0.62	0.21	0.23	0.01	0.01	0.01	0.01	0.22	0.22
	10	0.50	0.42	0.09	0.08	0.18	0.20	0.08	0.08	0.13	0.16	0.15	0.11
	13	0.58	0.49	0.60	0.50	0.48	0.52	0.50	0.54	0.19	0.24	0.50	0.10
	15	0.31	0.26	0.41	0.34	0.44	0.47	0.50	0.55	0.05	0.07	0.38	0.13
	17	0.73	0.62	0.06	0.05	0.01	0.01	0.29	0.31	0.035	0.03	0.05	0.22
		Samples											
		**M11**	**M12**	**M13**	**M14**	**M15**	**M16**	**M17**	**M18**	**M19**	**M20**	**Avarage**	**SD**
	1	0.05	0.04	0.01	0.02	0.06	0.08	0.12	0.07	0.03	0.02	0.05	0.03
	3	0.00	0.00	0.00	0.00	0.01	0.01	0.00	0.00	0.00	0.00	0.00	0.00
	6	0.01	0.01	0.01	0.01	0.07	0.08	0.01	0.01	0.01	0.01	0.01	0.02
**NS****	8	0.18	0.14	0.33	0.35	0.20	0.25	0.20	0.12	0.63	0.41	0.22	0.12
	10	0.16	0.13	0.27	0.28	0.14	0.17	0.27	0.16	0.28	0.18	0.18	0.06
	13	0.06	0.05	0.14	0.15	0.10	0.12	0.03	0.02	0.04	0.03	0.05	0.04
	15	0.23	0.19	0.36	0.38	0.34	0.42	0.44	0.27	0.40	0.26	0.35	0.07
	17	0.32	0.25	0.32	0.34	0.20	0.25	0.67	0.41	0.09	0.06	0.29	0.12

Continue

		Samples											
		**M21**	**M22**	**M23**	**M24**	**M25**	**M26**	**M27**	**M28**	**M29**	**M30**	**Avarage**	**SD**
	1	0.14	0.11	0.15	0.20	0.18	0.21	0.04	0.03	0.03	0.03	0.12	0.06
	3	0.03	0.02	0.00	0.00	0.45	0.52	0.00	0.00	0.00	0.00	0.01	0.18
	6	0.44	0.36	0.05	0.07	0.11	0.13	0.15	0.14	0.02	0.02	0.12	0.10
**VR*** **	8	0.45	0.37	0.09	0.12	0.40	0.47	0.31	0.29	0.37	0.36	0.36	0.10
	10	0.39	0.32	0.54	0.71	0.79	0.92	0.26	0.24	0.24	0.23	0.35	0.22
	13	0.56	0.46	0.40	0.53	0.48	0.56	0.22	0.20	0.52	0.50	0.49	0.10
	15	0.95	0.78	0.38	0.51	0.40	0.47	0.36	0.33	0.61	0.56	0.49	0.15
	17	0.76	0.62	0.25	0.33	0.26	0.31	0.56	0.52	0.47	0.43	0.45	0.13

*CO = control. packaging without tags; **NS: packaging with tags containing the nanosensor; ***VR = packaging with tags containing vermiculite absorber with a percentage of $KMnO_4$. simulating a commercial absorber; M = papaya;
